# A single-source precursor route to anisotropic halogen-doped zinc oxide particles as a promising candidate for new transparent conducting oxide materials

**DOI:** 10.3762/bjnano.6.222

**Published:** 2015-11-18

**Authors:** Daniela Lehr, Markus R Wagner, Johanna Flock, Julian S Reparaz, Clivia M Sotomayor Torres, Alexander Klaiber, Thomas Dekorsy, Sebastian Polarz

**Affiliations:** 1 Department of Chemistry, University of Konstanz, 78457 Konstanz, Germany; 2 ICN2 Catalan Institute of Nanoscience and Nanotechnology, Campus UAB, 08193 Bellaterra (Barcelona), Spain; 3 Department of Physics, University of Konstanz, 78457 Konstanz, Germany; 4 Catalan Institute of Research and Advanced Studies (ICREA), Barcelona 08010, Spain

**Keywords:** chemical doping, metal oxides, semiconductor nanoparticles, single-source precursors

## Abstract

Numerous applications in optoelectronics require electrically conducting materials with high optical transparency over the entire visible light range. A solid solution of indium oxide and substantial amounts of tin oxide for electronic doping (ITO) is currently the most prominent example for the class of so-called TCOs (transparent conducting oxides). Due to the limited, natural occurrence of indium and its steadily increasing price, it is highly desired to identify materials alternatives containing highly abundant chemical elements. The doping of other metal oxides (e.g., zinc oxide, ZnO) is a promising approach, but two problems can be identified. Phase separation might occur at the required high concentration of the doping element, and for successful electronic modification it is mandatory that the introduced heteroelement occupies a defined position in the lattice of the host material. In the case of ZnO, most attention has been attributed so far to n-doping via substitution of Zn^2+^ by other metals (e.g., Al^3+^). Here, we present first steps towards n-doped ZnO-based TCO materials via substitution in the anion lattice (O^2−^ versus halogenides). A special approach is presented, using novel single-source precursors containing a potential excerpt of the target lattice 'HalZn·Zn_3_O_3_' preorganized on the molecular scale (Hal = I, Br, Cl). We report about the synthesis of the precursors, their transformation into halogene-containing ZnO materials, and finally structural, optical and electronic properties are investigated using a combination of techniques including FT-Raman, low-*T* photoluminescence, impedance and THz spectroscopies.

## Introduction

There is an ever increasing demand for electrode materials exhibiting optical transparency in the visible region of the electromagnetic spectrum, because they are indispensable constituents in important technological devices such as flat panel displays, touch screens, solar cells and photocatalytic systems [1–4]. Among the different materials used for this purpose [5] such as carbon nanostructures [6–8], silver nanowires [9–10] or conducting polymers [11], the so-called TCOs (transparent conducting oxides) have some favorable features such as high durability, chemical resistance, e.g., against oxygen, water or other atmospheric contaminants, and they show good mechanical stability [1,12–13]. Optical transparency is realized by selection of a wide-gap semiconductor material, and substantial chemical doping leads to a sufficient amount of mobile charge carriers. The best-known example for TCOs is indium tin oxide (ITO) [4]. ITO can be characterized as a tin-doped indium oxide material with up to 90% content of In_2_O_3_. It is characterized by a low specific resistivity of the order ρ ≈ 1 × 10^2^ μΩ·cm. ITO is highly conductive, transparent in the visible and reflective in the IR spectral region [14]. Because indium is a rare element (with an abundancy of (1.00 × 10^−5^)% in the earth crust) [15], and due to the steadily growing need and price, there is a large demand for ITO substitutes [16].

TCOs consisting of chemically doped zinc oxide materials ('E@ZO'; E = heteroelement) have been recognized as promising candidates as ITO substitutes [17–18]. Because ZnO is already n-type in its native state, and due to the difficulties associated with p-doping [19–20], most attention was devoted to n-doping via substitution in the cation (Zn^2+^) lattice. The most prominent example known to date is aluminium-doped ZnO (AZO) [21–22]. AZO contains much more common elements compared to ITO, however, it cannot yet compete regarding its electrical properties. Much less work was invested in the preparation and investigation of n-doped ZnO via substitution in the anion lattice, which can be realized by the introduction of halogens instead of oxygen. There are only few papers describing F-doped ZnO (FZO) [23–25] and almost nothing is known about Cl@ZnO [26–30], Br@ZnO and I@ZnO. This is because successful lattice substitution becomes more difficult, the larger the difference between the ionic radius of Hal^−^ (*r*(F^-^) = 131 pm; *r*(Cl^−^) = 181 pm; *r*(Br^−^) = 196 pm, *r*(I^−^) = 220 pm) and O^2−^ (*r*(O^2−^) = 138 pm) is.

Physical methods such as different variants of physical vapor deposition and sputtering have been proven to be a good technique for the preparation of thin films of E@ZnO on different substrates [31–34]. Despite some inherent advantages such as good crystallinity of the materials, some drawbacks of the mentioned physical methods are that they require quite demanding, high-vacuum equipment, correct process parameters are often difficult to find, and nanostructures different from thin films cannot be obtained. For the preparation of semiconductor nanomaterials such as colloidal particles, quantum dots or porous materials bottom-up synthesis routes in the liquid phase are commonly applied [35–37]. Whereas, bottom-up techniques such as the sol–gel process for metal oxides [38–39] work perfectly for the generation of an entire zoo of nanostructures, to realize at the same time intentional doping of those nanostructures is extremely demanding [40]. Often a desired heteroelement does not incorporate, e.g., due to different solubility in the used solvent or due to different reaction kinetics. It can be concluded, that there is currently an important deficiency between the potential of physical methods granting precisely doped semiconductors and the potential of bottom-up methods enabling control over materials morphology.

The mentioned problem could be solved, if one uses special molecules for materials synthesis. So-called molecular single-source precursors (MSSPs) contain, on the molecular level, all required elements for the final material [41–43]. They are very reactive, guarantee a high mass transport and can be converted into the solid material at relatively low temperatures. Whereas a substantial number of MSSPs for binary materials are known, there are only few examples for ternary phases as targets [44–46]. Our group has extensive experience with MSSPs for ZnO materials [47–49], and several examples for ternary phases (Mn@ZnO, Ni@ZnO, Cr@ZnO, Li@ZnO, S@ZnO) were reported [50–54]. The advantage of using MSSPs for ternary phases is, that kinetically controlled synthesis routes can be realized leading to very high substitution degrees, in some cases even above the solubility limit.

In the current contribution, we are seeking for a novel MSSP route towards Hal@ZnO materials. First, we will describe the synthesis and characterization of the molecular precursors. Then, their transformation into Hal@ZnO materials will be studied. Focusing on Cl@ZnO, important optical and electronic properties will be investigated in detail.

## Experimental

### Synthesis

All starting compounds were received from Aldrich, were purified and carefully dried prior to use. All reactions were performed under strict exclusion of air and humidity using Schlenk techniques. [MeZnO*t*-Bu]_4_ and [EtZnOiPr]_4_ were synthesized according to the literature [55].

**Preparation of [I(CH_3_)_3_Zn_4_(O*t*-Bu)_4_] (2a):** In 60 mL toluene 4 g [MeZnO*t*-Bu]4 (6.51 mmol) were dissolved and cooled to −78 °C. A solution of 1.65 g dried I_2_ in 40 mL toluene was added drop-wise. The solution was warmed up to room temperature overnight under continuous stirring. The solvent was removed under reduced pressure. The residue was dissolved in 40 mL hexane and insoluble impurities were removed via centrifugation. The solvent was removed under reduced pressure and the product was obtained as a colorless solid (78%). ^1^H NMR (400 MHz, CDCl_3_) δ −0.37 (s, 1H, ZnC*H*_3_), 1.39 (s, 1H, C*H*_3_), 1.48 ppm (s, 3H, C*H*_3_); ^13^C NMR (100 MHz, CDCl_3_) δ −6.66 (Zn*C*H_3_), 32.11 (C(*C*H_3_)_3_), 32.29 (C(*C*H_3_)_3_), 75.15 (*C*(CH_3_)_3_), 76.12 (*C*(CH_3_)_3_); MS (EI) *m*/*z*: *M*-CH_3_^+^ 710.9.

**Preparation of [Br(CH_3_)_3_Zn_4_(O*t*-Bu)_4_] (2b):** In 60 mL hexane 3 g [MeZnO*t*-Bu]4 (6.51 mmol) were dissolved and cooled to −78 °C. 4.9 mL of 1M Br_2_ solution in hexane were diluted with 18 mL hexane and added dropwise. The solution was warmed up to room temperature overnight under continuous stirring. Insoluble impurities were removed via centrifugation; the solvent was removed under reduced pressure and the product was obtained as a colorless solid (52%). ^1^H NMR (400 MHz, CDCl_3_) δ −0.38 (s, 1H, ZnC*H*_3_), 1.38 (s, 1H, C*H*_3_), 1.45 ppm (s, 3H, C*H*_3_); ^13^C NMR (100 MHz, CDCl_3_) δ −7.16 (Zn*C*H_3_), 32.09 (C(*C*H_3_)_3_), 32.13 (C(*C*H_3_)_3_), 75.08 (*C*(CH_3_)_3_), 75.88 (*C*(CH_3_)_3_).

**Preparation of [ClEt_3_Zn_4_(OiPr)_4_] (2c):** 3.5 g [EtZnOiPr]_4_ (5.7 mmol) were dissolved in 70 mL toluene and cooled to −78 °C. 5.7 mL of 1 M HCl·Et_2_O were diluted with 16 mL Et_2_O and added dropwise. The solution was warmed up to room temperature overnight under continuous stirring. Insoluble impurities were removed via centrifugation; the solvent was removed under reduced pressure. The colorless solid was recrystallized from hexane (56%). ^1^H NMR (400 MHz, CDCl_3_) δ 0.37 (q, ^3^*J* = 8.1 Hz, 6H, ZnC*H*_2_CH_3_), 1.26 (t, ^3^*J* = 8.1 Hz, 9H, ZnCH_2_C*H*_3_), 1.30, 1.35 (each d, ^3^*J* = 6.1 Hz, altogether 24H, C*H*_3_), 4.24, 4.17 (each h, ^3^*J* = 6.1 Hz, altogether 4H, C*H*) ppm; ^13^C NMR (100 MHz, CDCl_3_) δ 1.28 (Zn*C*H_2_CH_3_), 12.20 (ZnCH_2_*C*H_3_), 27.05 (CH(*C*H_3_)_2_), 27.14 (CH(*C*H_3_)_2_), 69.29 (*C*H(CH_3_)_2_), 69.74 (*C*H(CH_3_)_2_).

**Preparation of Hal@ZnO**: The single-source-precursor **2a**, **2b** or **2c** is placed in a porcelain combustion boat and transferred into a tube oven (Nabertherm R50/500/12, equipped with a 2.7 cm diameter quartz tube). Under N_2_/O_2_ atmosphere (0.05 L/min N_2_, 0.2 L/min O_2_) the oven was heated up (heating rate 2 K/min) to 350 °C and the temperature was kept for 10 h.

**Preparation of ZnO_1−_*_x_*Cl*_x_* with various doping levels:** As an example 1.0 g [EtZnOiPr]_4_ (1.63 mmol) and 0.088 g [ClEt_3_Zn_4_(OiPr)_4_] (0.14 mmol) were dissolved in 20 mL THF. After stirring for 10 min, the solvent was removed under reduced pressure. The precursor mixture was placed in a porcelain combustion boat and transferred into a tube oven (Nabertherm R50/500/12, equipped with a 2.7 cm diameter quartz tube). Under N_2_/O_2_ atmosphere (0.05 L/min N_2_, 0.2 L/min O_2_) the oven was heated up (heating rate 2 K/min) to 350 °C and the temperature was kept for 10 h. Materials with smaller chlorine content were obtained by decreasing the amount of [ClEt_3_Zn_4_(OiPr)_4_] to 0.064 respectively 0.051 g in the precursor mixture. A ZnO reference was synthesized from pure [EtZnOiPr]_4_ precursor under the same reaction conditions.

### Analytical techniques

NMR spectra were acquired on a Bruker Avance III spectrometer. X-ray diffraction was performed on a Bruker AXS D8 Advance diffractometer using Cu Kα radiation. Raman measurements were conducted on a Horiba LabRAM HR spectrometer using a 532 nm DPSS laser and a 100× microscope objective. Room temperature PL measurements were performed on a Horiba Fluorolog spectrometer. The UV–vis measurements were done on a Varian Cary 100 scan UV–vis spectrophotometer equipped with an Ulbricht reflecting sphere. TGA analysis was performed on a Netzsch STA 449F3. EDX spectra were acquired on a Hitachi TM 3000 SEM equipped with a Bruker Quantax 70 detector. Dielectric measurements were performed with a Zahner IM6. For each sample 40 mg of ZnO_1−_*_x_*Cl*_x_* powder were pressed to disc-shaped pellets with a diameter of 0.8 cm shortly before measurement. The pellets were sandwiched between disc-shaped electrodes (0.8 cm diameter) and measured under dry conditions (glove box) one after another. Micro-Raman and low-temperature photoluminescence measurements were performed using a Horiba Jobin Yvon T64000 setup with a nitrogen-cooled CCD. Low-temperature photoluminescence measurements were conducted using an Oxford micro-cryostat with a temperature range from 4 to 350 K. The samples were excited by the 350 nm line of a krypton ion laser with an excitation power of no more than 500 μW to avoid heating by UV absorption. Raman spectra were acquired using the 514 nm lines of an argon ion laser with an excitation power of 3 mW. In both cases, an Olympus 50× microscope objective was used to focus and collect the emitted and scattered light from the sample. Time-domain THz spectroscopy was performed with a custom-build transmission setup employing asynchronous optical sampling (ASOPS) with two frequency-locked femtosecond Ti:sapphire lasers with repetition rates of one GHz [56–57]. The data were partially evaluated with the commercial software TeraLyzer. The DFT calculations have been carried out using the def2-TZVP basis set for all atoms and B3LYP functional.

## Results and Discussion

### Precursor synthesis

**Scheme 1:**
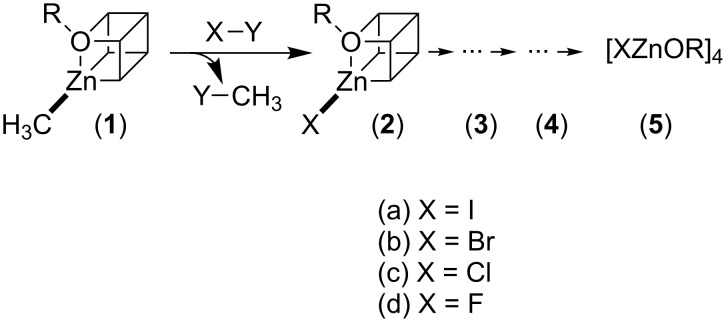
Synthesis of halogen-substituted alkylzinc–alkoxide precursors.

Alkylzinc–alkoxides with heterocubane structure [CH_3_ZnOR]_4_ are well known precursors for the synthesis of various ZnO materials [58–63]. It is documented that the reaction with water proceeds via the stepwise elimination of methane and the formation of hydroxo zinc species prior to polycondensation affording zinc oxide [55]. The latter process can also be interpreted as the reaction between H^δ+^–OH as a Lewis and Brønsted acid and CH_3_^δ−^–Zn as a Brønsted base accompanied by metathesis. Therefore, the question arises if the treatment of [CH_3_ZnOR]_4_ with alternative Lewis (X–Y) acids under non-aqueous conditions could lead to defined species, partially substituted at the Zn^2+^ position (see Scheme 1). In the current paper, we consider as potential Lewis acids either elemental halogens (X = Hal, Y = Hal) [64] or mineral acids (Y = H, X = Hal). An advantage of this approach is that the by-product CH_3_Y (Y = H, Hal) is highly volatile and can be removed from equilibrium very easily.

**Figure 1:**
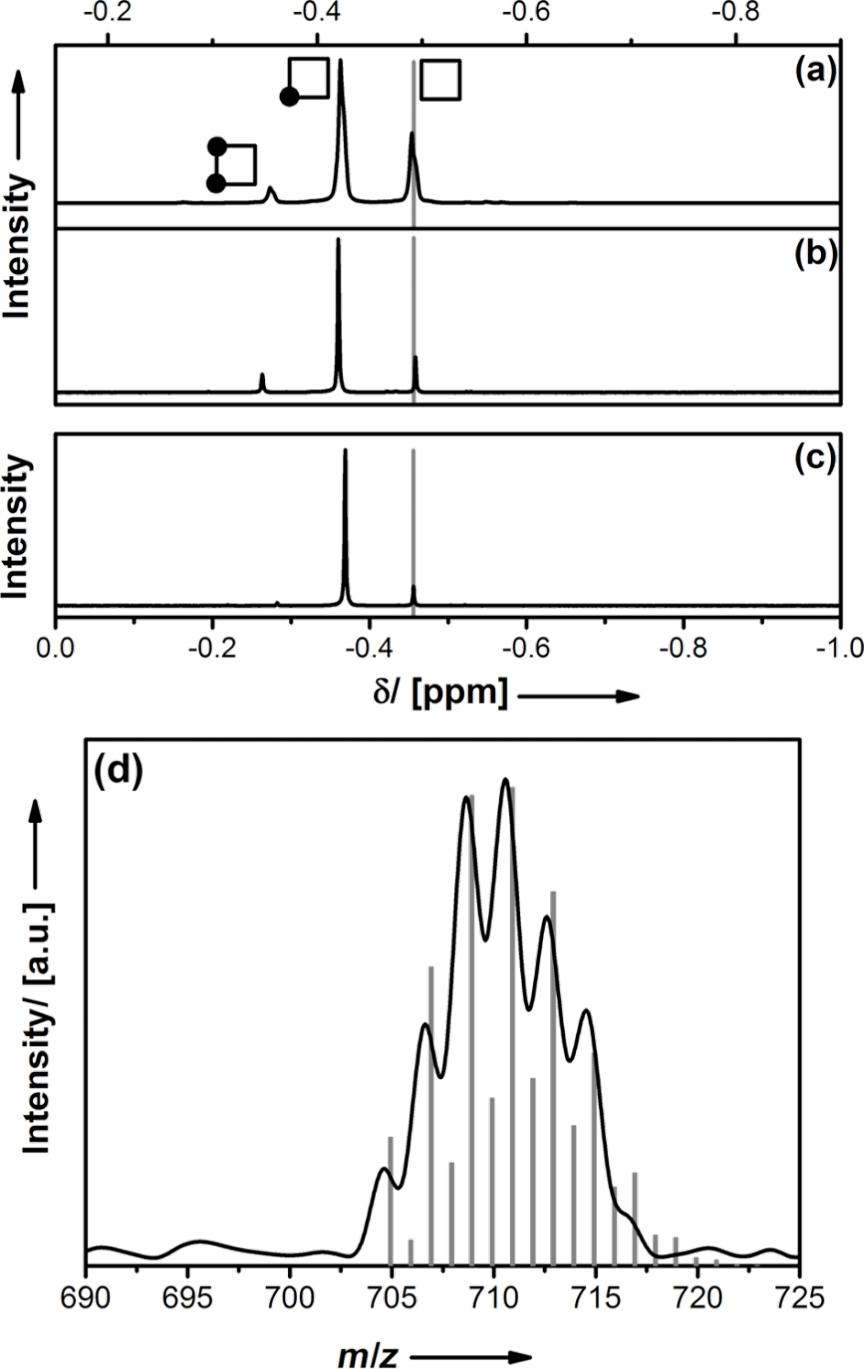
^1^H NMR spectra (Zn–CH_3_ region) for the reaction of I_2_ with [MeZnOiPr]_4_ before (a) and after purification (b), and for the reaction with [MeZnO*t*-Bu]_4_ (c). The assignment of the signals to different ZnMe → ZnI substitution degrees is shown schematically (a). The grey bar indicates the peak for the respective starting compound **1**. (d) Excerpt from the EI-MS spectrum (black line) of compound **2a** and calculated signals for the fragment [I(CH_3_)_2_Zn_4_(O*t*-Bu)_4_]^+^ (*m*/*z* = 710.9).

A solution of [MeZnOiPr]_4_ or [MeZnO*t*-Bu]_4_ was reacted with I_2_ in toluene at *T* = −78 °C (see Section Experimental). One sees an immediate decoloration of the solution, indicating the consumption of iodine. Nuclear magnetic resonance (NMR) and in particular ^1^H NMR is well suited for the analysis of the obtained product distribution (Figure 1). Because the electronegativity of iodine (χ = 2.66) is slightly higher than the group negativity of methyl (χ = 2.31) [65], one sees a shift of the signals for Zn–CH_3_ entities to lower field, the more Zn–I units are already present in the heterocubane structure. The reaction using [MeZnOiPr]_4_ leads to a mixture of the starting compound **1**, containing the monosubstituted product [I(CH_3_)_3_Zn_4_(OiPr)_4_] (**2a**) (55%) and the twofold-substituted product [I_2_(CH_3_)_3_Zn_4_(OiPr)_4_] (**3a**) (10%) (Figure 1a). Purification via recrystallization for several times is able to remove substantial parts of the starting compound **1**, but the relative amount of **3a** remains unchanged. Obviously, iodine reacts too fast with **1**, which makes double substitution possible. The possibility for the second substitution could be reduced by decreasing the overall concentration of the reactants and by selecting the less reactive *tert*-butyl compound [MeZnO*t*-Bu]_4_. It can be seen that the monosubstituted compound [I(CH_3_)_3_Zn_4_(O*t*-Bu)_4_] (**2a**) could now be obtained in satisfactory purity (Figure 1c). The substitution with iodine also changes the symmetry of the molecular compound (T_d_ → *C*_3_*_v_*). Whereas all *tert*-butoxy groups in **1** are symmetry equivalent resulting in one distinct signal in ^1^H and ^13^C NMR, there are now two different *tert*-butoxy groups with a ratio of 3:1 in **2a** and slightly different chemical shifts (Figure S1, Supporting Information File 1). Further evidence of the molecular structure results from comparison and analysis of the FT-Raman spectra of **1** and **2a** (see Figure S1, Supporting Information File 1). It is clearly visible that all vibration modes of the starting compound are still present in the Raman spectra **2a**, which is an indication that the heterocubane framework is still intact. Additionally a sharp signal occurs at 170 cm^−1^, which can be assigned to the Zn–I vibration (expected at 166 cm^−1^).

Single crystals of **2** were grown for X-ray diffraction analysis. Unfortunately, the exact structure determination was not possible due to substantial orientational disorder. The heterocubane core and the presence of iodine could be confirmed, but a satisfactory solution of the crystallographic data was not possible. Obviously, despite the lower symmetry, still all different orientations of the heterocubane in the crystal lattice were possible, leading to a randomized positioning of Zn–I on symmetry equivalent positions. Therefore, independent analytical techniques were required for proving that the proposed molecular compound could be obtained. The most intense signal in the electron-impact mass spectrum (EIMS) of compound **2a** is shown in Figure 1d. The observation of the [I(CH_3_)_2_Zn_4_(O*t*-Bu)_4_]^+^ fragment clearly indicates the success of our synthetic route.

A bromo-substituted compound [Br(CH_3_)_3_Zn_4_(O*t*-Bu)_4_] (**2b**) could be obtained in an analogous way using Br_2_ as a reactant and [MeZnO*t*-Bu]_4_ as starting compound (see Figure S2, Supporting Information File 1). Due to the high reactivity of elemental bromine, the reaction leads to a mixture of the starting compound, the monosubstituted product [Br(CH_3_)_3_Zn_4_(O*t*-Bu)_4_] (**2b**) (73%) and small amounts of the twofold-substituted product [Br_2_(CH_3_)_2_Zn_4_(O*t*-Bu)_4_] (**3**) (6%). However, when **1** was treated with Cl_2_, a complex mixture of different compounds was obtained, and purification was not possible. Therefore, alternative routes towards the desired monochlorinated compound needed to be explored. The reactivity of the starting, heterocubane compound could be reduced further, when using ethylzinc instead of the methylzinc derivative [55]. Furthermore, the dipolar character of the Zn–CH_2_CH_3_ bond in [EtZnOiPr]_4_ was now addressed by using hydrogen chloride etherate HCl·Et_2_O as a reactant (see Section Experimental). NMR, FT-Raman and EIMS data showed (see Figure S3, Supporting Information File 1) that the desired compound [Cl(Et)_3_Zn_4_(OiPr)_4_] has been obtained in sufficient purity. Unfortunately, the preparation of an analogous compound containing fluorine, e.g., [F(Et)_3_Zn_4_(OiPr)_4_] as a potential single-source precursor for F-doped ZnO remained an unsolved challenge for us up to this point. Conventional fluorinating agents such as Me_3_SnF, NEt_3_·3HF, Bu_4_N^+^F^−^ or Bu_4_N^+^PF_6_^−^ either seem to be not reactive enough, or they contain irremovable water, which leads to an undesired reaction of the heterocubane precursor to ZnO via sol–gel chemistry [66–67]. Elemental fluorine and anhydrous HF have not been tested yet, because of security issues handling these compounds.

### Materials synthesis and properties

**Figure 2:**
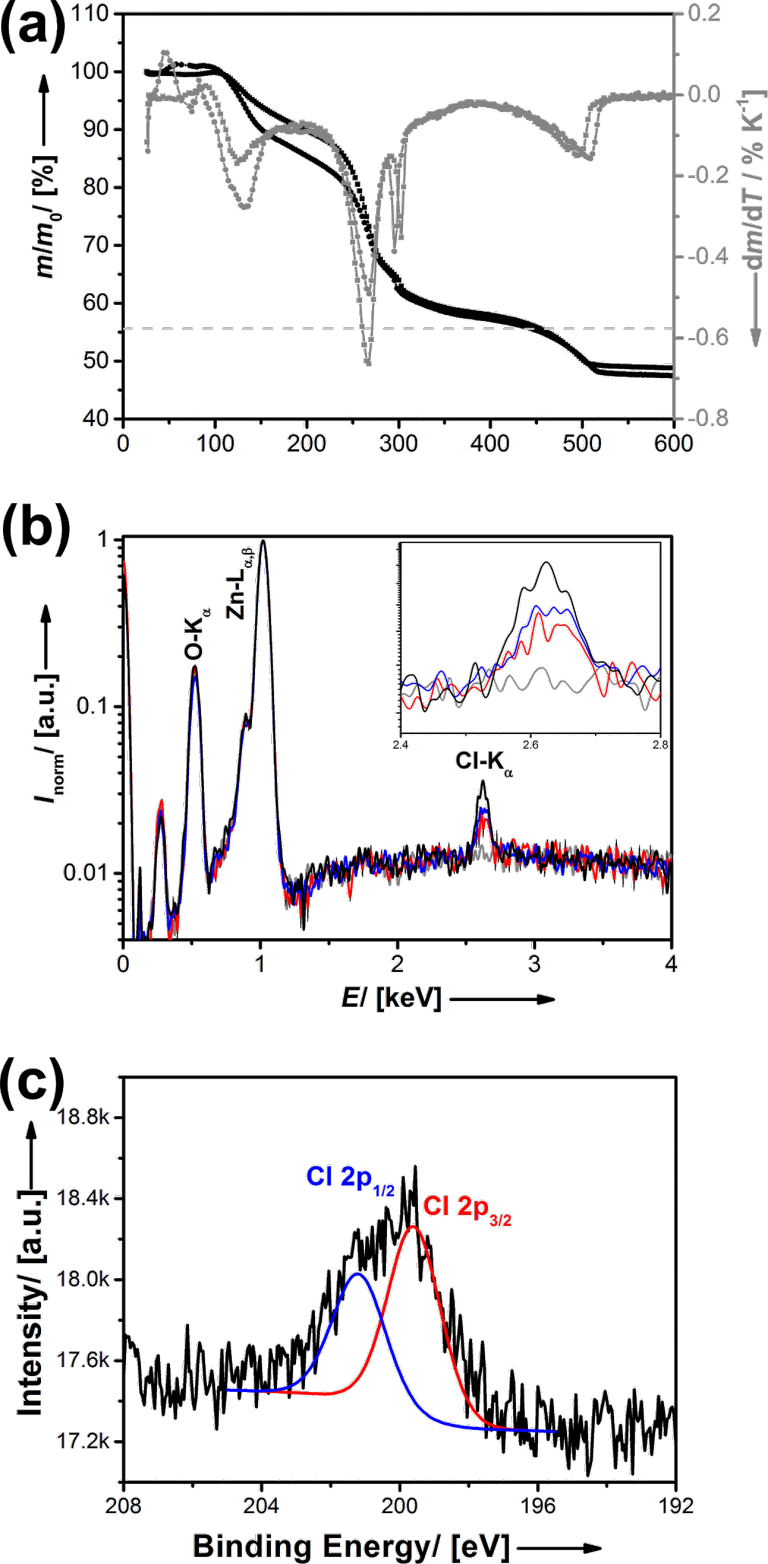
(a) TGA traces (black) and its first derivative (grey) of the thermal decomposition of the molecular precursor compound [Cl(Et)_3_Zn_4_(OiPr)_4_] in nitrogen atmosphere (squares) and artificial air (circles), heating rate: 5 K/min. The dashed grey line indicates the remaining mass expected for complete removal of any organic constituents. (b) EDX spectra for pure ZnO as a reference (grey graph) and ZnO_1−_*_x_*Cl*_x_* materials with different chlorine content: 3.6% (black graph), 1.8% (blue graph) and 1.4% (red graph). (c) XPS spectrum for the Cl-2p region.

Given the potential precursors for Cl@ZnO, Br@ZnO, and I@ZnO, it should be noted that the difference in the ionic radius compared to O^2−^ is smallest for Cl^−^ in the given series (Δ*r* = +38%). Therefore, we present the generation of chlorine containing zinc oxide first and discuss the results in detail. We chose a thermal route for the transformation of the precursors into the final materials. Thermogravimetric analysis (TGA) reveals important information about the thermal behavior of the anticipated single source precursor [Cl(Et)_3_Zn_4_(OiPr)_4_] (Figure 2a). It can be seen that there is hardly any difference between the TGA traces in inert atmosphere (N_2_) and in air (20% O_2_), which indicates that oxygen is not pivotal for the complete transformation of the precursor. The latter is an important criterion for a single-source precursor, and an advantage because oxygen can also react with halogens at higher temperature and lead to an undesired exchange. Four distinct mass loss steps can be identified from the first derivative of the TGA curve (Figure 2a). Considering the associated values for Δ*m*, one can conclude that the first step (*T* = 131 °C) is caused by the loss of the ethyl groups attached to zinc. Then, the isopropyl residue leaves the molecule as propene (*T* = 265 °C). The remaining mass for 400 °C < *T* < 450 °C fits very well to the residual mass expected for the complete conversion of the precursor into a non-charged product (*m*/*m*_0_ = 55.6%): 2 × (**2c**)→'ZnCl_2_·(ZnO)_7_'.

**Figure 3:**
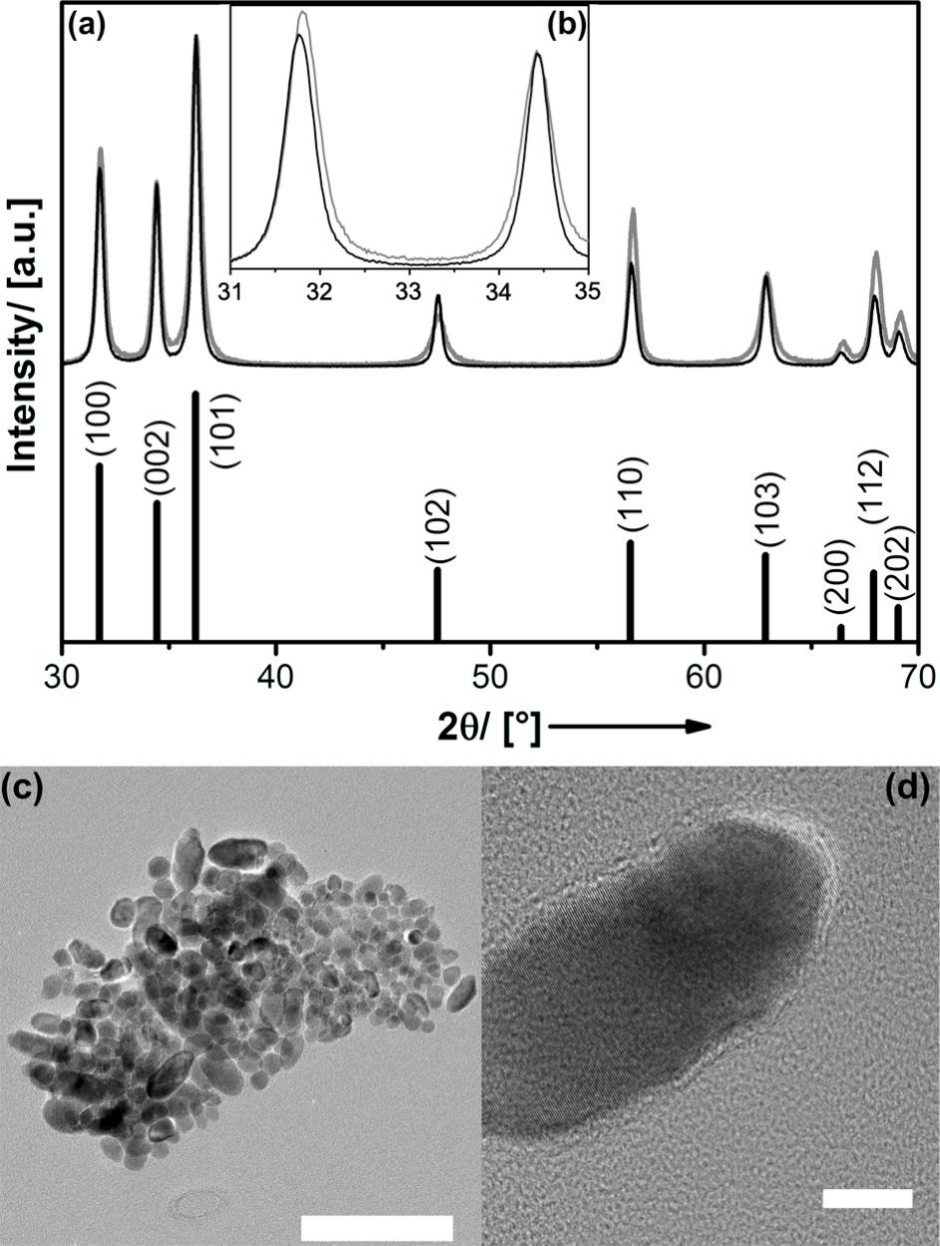
(a) Experimental PXRD pattern for materials prepared via thermolysis of [Cl(Et)_3_Zn_4_(OiPr)_4_] (black graph) and [EtZnOiPr]_4_ (grey graph). The pattern of ZnO (wurtzite) is shown as black bars. (b) Enlargement of the 2θ region 31–35° for better visibility of the (100) and (002) diffraction signals. (c) TEM (scalebar 100 nm) and HRTEM (scalebar 10 nm) of the ZnO_1−_*_x_*Cl*_x_* material. See also Figure S4 (Supporting Information File 1).

There is an additional and final step of mass loss at *T* = 450 °C (Δ*m* ≈ 10%), which fits to the amount of chlorine present in the precursor. Therefore, one can assume that chlorine is removed very rapidly from the material at temperatures above 500 °C, which can also be confirmed by EDX. Obviously, it is very important to prepare the materials at lower temperatures to make sure that Cl remains as a dopant in the ZnO host. Therefore, Cl@ZnO was synthesized via thermal decomposition of **2c** for 10 h at lower temperature (350 °C) under N_2_/O_2_ atmosphere. The presence of Cl in the obtained material could be investigated by selected-area energy dispersive X-ray spectroscopy (EDX) (Figure 2b) and X-ray photoelectron spectroscopy (Figure 2c). The maximum concentration of Cl in the ZnO lattice we could reach via this method is 3.6 atom % (≡ZnO_0.964_Cl_0.036_). The low chlorine values compared to the molecular precursor shows that even at lower temperature, there is a slow but steady loss/evaporation of chlorine. Higher chlorine values of up to 10 atom % could be realized for shorter periods of thermal treatment or for sacrifice of oxygen, but then the sample is contaminated with residual carbon. Because the amount of chlorine is still very high, a potential phase separation into ZnO and ZnCl_2_ could be a problem. Therefore, the material prepared at *T* = 350 °C was investigated using powder X-ray diffraction (PXRD) analysis shown in Figure 3a.

As a reference pure ZnO materials were prepared under analogous conditions, but using [EtZnOiPr]_4_ as a precursor, which yields pure, nanocrystalline ZnO with wurtzite structure (space group *P*6_3_*mc*, in agreement to previous studies) [49,63]. Also with [Cl(Et)_3_Zn_4_(OiPr)_4_] as a precursor there are no diffraction signals other than those for wurtzite. However, there are some subtle differences (Figure 3b). The PXRD patterns were normalized to the intensity of the (002) signal. It can be seen that the intensity of the (100) signal of the chlorine containing material is smaller than that of the pure ZnO material, which suggests that the aspect ratio (extension in direction of the crystallographic *c*-axis/*a*,*b*-extension) has become slightly larger due to the presence of chlorine. This can be confirmed by transmission electron microscopy (TEM) investigations shown in Figure 3c and Figure S4 (Supporting Information File 1).

The particle size distribution is polydisperse and almost all particles have an anisotropic, elongated shape. In contrast, the particles of the pure ZnO material are not elongated (see Figure S5, Supporting Information File 1). High-resolution (HR) TEM measurements show the high crystallinity of the single Cl@ZnO nanocrystals (see also Figure S4, Supporting Information File 1), the lattice plane distance seen in Figure 3d is *d* = 0.2618 nm. The comparison to *d*_002_ = 0.2604 nm of pure ZnO indicates that the main growth direction of the particles is indeed the crystallographic *c*-axis. It is interesting to note that according to the shift of the respective PXRD signals (2θ_100_, 2θ_002_) (Figure 3b) the incorporation of Cl^−^ leads to a widening of the lattice in the crystallographic *a*,*b*-direction (Δ*a* = Δ*b* = 0.146 Å) but not in *c*-direction. Thus, a potential explanation for the morphological influence of chlorine doping could be that the average density of surfaces corresponding to lattice planes with *a*,*b* components will be decreased. The latter will raise the surface energy and also the apposition rate of ZnO species on the surfaces [68]. As a result main growth takes places perpendicular to *a*,*b* (Figure 3d). The hypothesis that the presence of Cl also influences the growth rate could be confirmed by preparing a series of ZnO_1−_*_x_*Cl*_x_* samples differing systematically in chlorine content. The materials were prepared from molecular precursor mixtures of [EtZnOiPr]_4_ and [ClEt_3_Zn_4_(OiPr)_4_], the required doping concentration was adjusted via the precursor composition (see Section Experimental). Nevertheless, the chlorine content determined experimentally in ZnO_1−_*_x_*Cl*_x_* (*x* = 0, 0.014, 0.018, 0.025) differs slightly from that in the precursor mixture (*x* = 0, 0.0125, 0.015, 0.02). This is because of sublimation of the [EtZnOiPr]_4_ precursor and weighing inaccuracies. The ZnO_1−_*_x_*Cl*_x_* samples were analyzed by PXRD and the results are shown in Figure S6 (Supporting Information File 1). It can be seen that with higher content of chlorine (under otherwise constant conditions) also the particle size increases.

**Figure 4:**
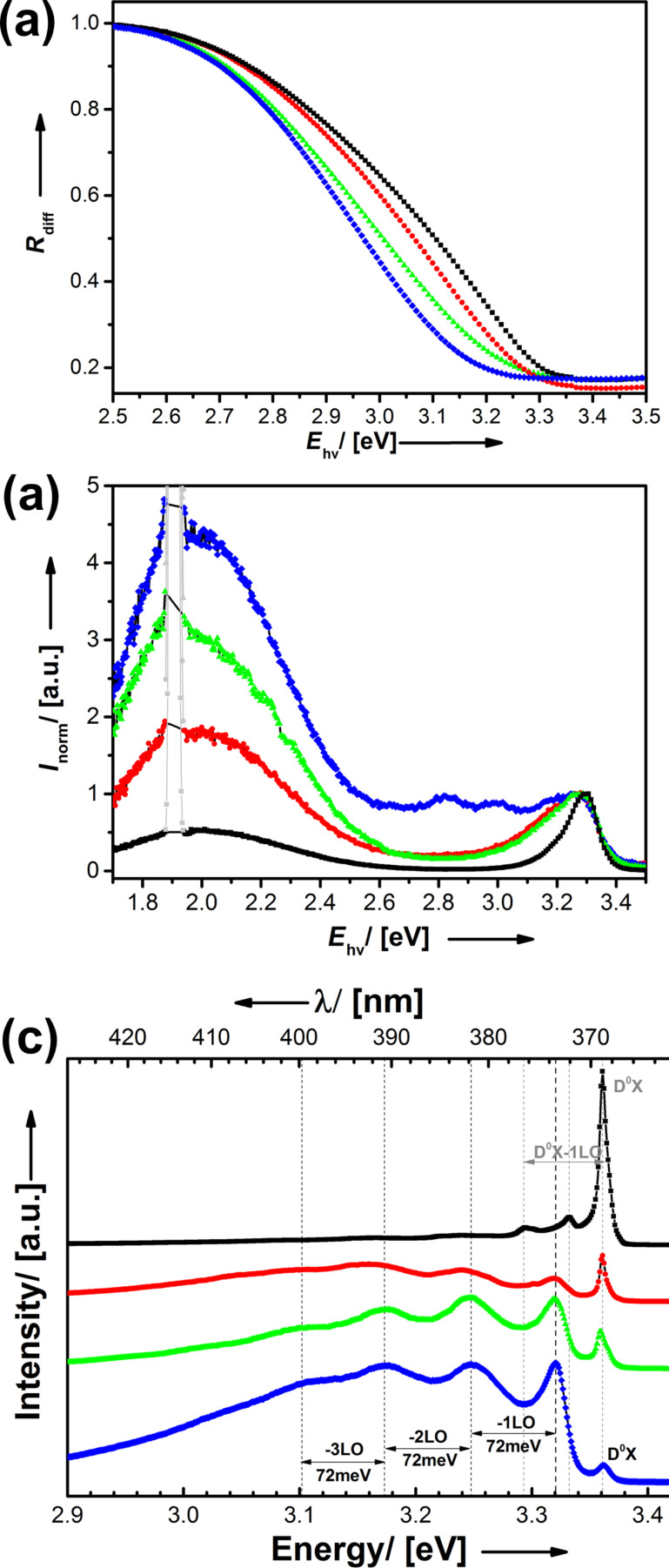
(a) Absorption spectra in diffuse reflection modus, room temperature photoluminescence spectra; overview (b) and band gap region (c). (d) PL spectra recorded at *T* = 7 K. Hashes (blue): ZnO (*D*_cryst_ = 22 nm); triangles (green): ZnO_0.986_Cl_0.014_ (*D*_cryst_ = 25 nm); circles (red): ZnO_0.982_Cl_0.018_ (*D*_cryst_ = 27 nm); squares (black): ZnO_0.964_Cl_0.036_ (*D*_cryst_ = 37 nm).

Optical spectroscopy is typically a good tool to gather first information about some electronic properties of semiconductor compounds. Absorption spectra were acquired in diffuse reflection mode (*R*_diff_) and are shown in Figure 4a for ZnO_1−_*_x_*Cl*_x_* samples. It can be seen that chlorine doping leads to a shift of the band gap energy, *E*_gap_, to higher energies, and there is a linear correlation of the blue shift with Cl content (see Figure S6b, Supporting Information File 1). Because in this series also the crystallite size increases with χ_Cl_, it can be ruled out that the quantum size effect is responsible for the observed shift. Another explanation could be that the incorporation leads to strain.

However, this is not in agreement with PXRD data, FT-Raman data (see below) and low-*T* photoluminescence measurements. Therefore, it seems that the enlargement of *E*_gap_ is due to electronic reasons. For semiconductors with high doping and a significant concentration of free charge carriers one knows the so-called Burstein–Moss effect [69–70]: Valence-band electrons need to be excited into vacant states at higher energies due to a substantial population of Bloch functions near the lower edge of the conduction band. As a consequence the band gap gets overestimated. It should also be noted, that the measurements show a high transparency in the visible region (≈2.7–1.7 eV). However, whether the free carrier concentration of the materials presented is here is sufficiently high, is a matter of discussion.

The described blue shift can be confirmed by photoluminescence (PL) spectroscopy recorded at room temperature (Figure 4c). Furthermore, Cl doping strongly affects the so-called green luminescence, which is associated with electrons trapped at oxygen vacancies (V_O_•• in Kröger–Vink notation) present in many different types of ZnO materials [63]. It can be seen that the intensity of the green luminescence (normalized to the near band-edge emission) gradually decreases with increasing amount of chlorine (Figure 4b). The latter observation might be explained as follows. Substitution of O^2−^ by Cl^−^ leads to an excess of positive charges in the lattice (Cl_O_•). Thus, point defects that also lead to relative positive charges such as V_O_•• become more and more unfavorable and the green luminescence is suppressed. The low-temperature photoluminescence spectra measured at 7 K (Figure 4d) provide detailed information about the near band-edge luminescence. The spectrum of the undoped ZnO sample is dominated by a transition at 3.32 eV. This zero-phonon line is repeated by longitudinal optical phonon replicas with an energy spacing of 72 meV. The high intensity of the phonon replicas is characteristic for a strong electron–phonon coupling as it is typically observed for donor acceptor pairs (DAP) [71], although the energy is closer to the 3.31 eV free-to-bound transition reported by Schirra et al. [72]. With increasing Cl concentration the signal disappears. At the same time a transition, which can be assigned to bound exciton (D^0^X), becomes more intensive. The spectral position coincides with typical shallow donor bound excitons in the neutral charge state and is found very close to the well-known position of the shallow Ga donor D^0^X I_8_ or Al D^0^X I_6_ [73]. In addition an emission line at 3.333 eV is visible in the spectra of ZnO_1−_*_x_*Cl*_x_* with *x* = 2.5%. This transition, which is labeled Y_0_, is assigned to a donor-bound exciton recombination at a structural defect [74]. The spectra of the 2.5% sample are dominated by the D^0^X shallow donor-bound exciton recombination with one phonon replica of the D^0^X visible. The low-temperature PL spectrum of the highest doped Cl@ZnO sample resembles those of good quality ZnO single crystals. Once again the PL measurements demonstrate that the crystal quality of ZnO materials prepared from molecular precursors can be improved by controlled Cl doping. The synthetic strategy via molecular precursors enables not only a high doping concentration, but also the access to ZnO with a low defect density.

**Figure 5:**
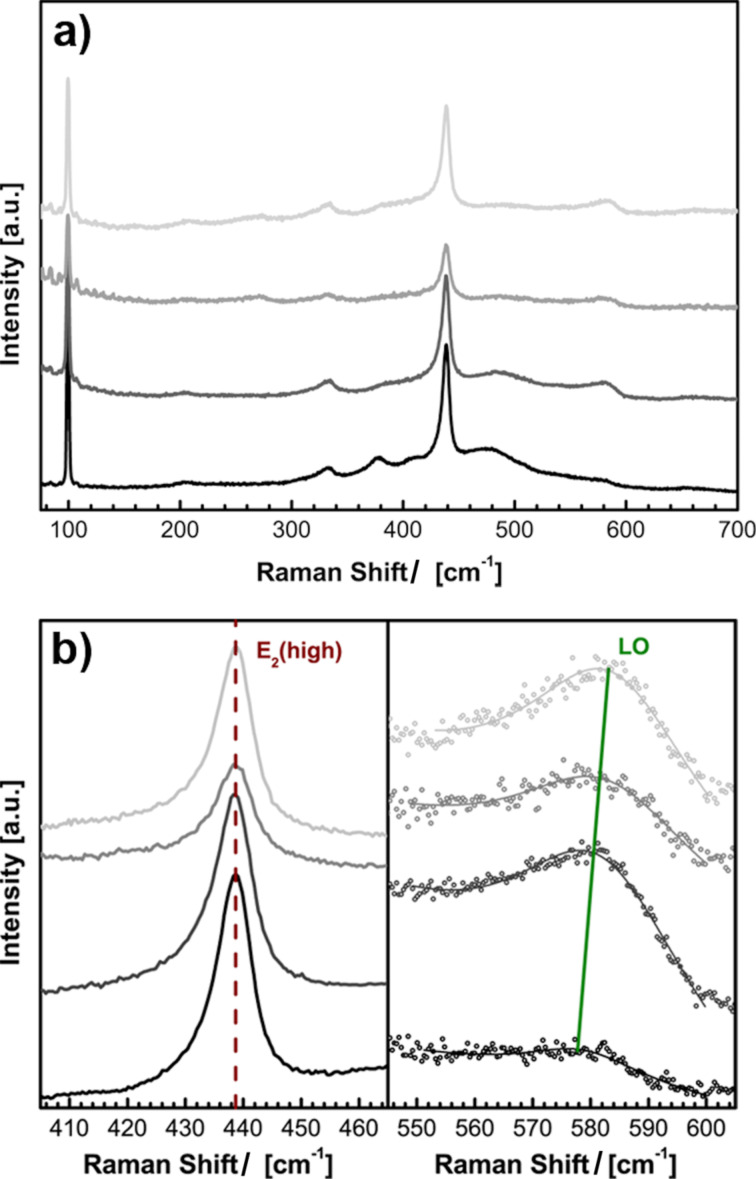
(a) Raman spectra of ZnO_1−_*_x_*Cl*_x_*: *x* = 0.0% (black), 1.4% (dark grey), 1.8% (grey) and 2.5% (light grey); (b) Raman spectra in the range of the non-polar E_2_ (high) and polar LO modes.

Further information can be acquired by vibrational spectroscopy. Raman spectroscopy is a powerful method to investigate structural and electronic properties of semiconductors [75]. The non-polar E_2_^high^ mode of hexagonal wurtzite ZnO is very sensitive to lattice strain [76]. The LO mode can interact with electrons from the conduction band, the magnitude depends on the free carrier distribution [77–78]. The Raman spectra shown in Figure 5 are dominated by the characteristic vibrational modes E_2_^low^ at about 98 cm^−1^ and E_2_^high^ at about 439 cm^−1^ [79].

The spectral position of the E_2_^high^ mode remains constant with increased doping concentration, which proves that the Cl doping does not affect strain in the ZnO lattice (Figure 5b). The spectral position of the LO mode is shifted to higher wavelengths with increased Cl doping. This effect is based on longitudinal optical phonon–plasmon coupling and describes the interaction of collective oscillating free carriers (plasmons) with LO phonons [80].

**Figure 6:**
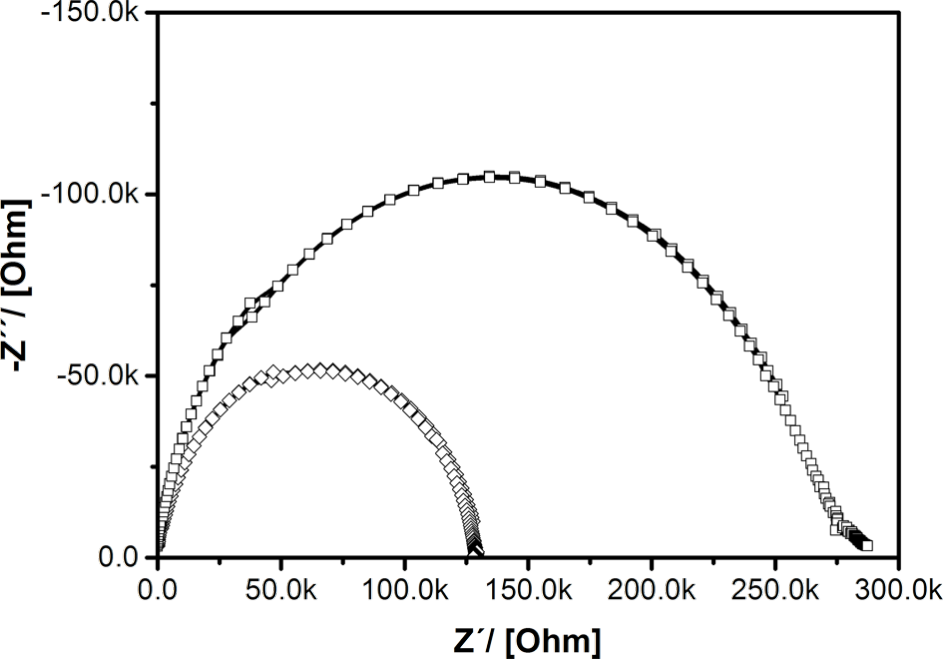
Impedance spectra (Nyquist Plots) of ZnO_0.986_Cl_0.014_ (hashes) and pure ZnO (squares) prepared in an analogous way.

Consequently, the concentration of free carriers increases with Cl doping. The dielectric properties of thin ZnO_1−_*_x_*Cl*_x_* pellets were investigated with impedance spectroscopy. In the Nyquist plot in Figure 6 the imaginary part of the impedance is plotted as a function of the real part. For materials having resistive and capacitive components a series of two semicircles usually occurs in the Nyquist plot. The semicircle in the high frequency region is assigned to grain contribution while the semicircle in the low frequency region is assigned to grain boundary contribution [81]. Because only one single semicircle at low frequencies is present in Figure 6, it can be concluded that grain-boundary resistance dominates over grain resistance. The resistance values of the Cl@ZnO pellets are obtained from the circular arc intercepts on the *Z*′-axis. For *x* = 1.4% the resistance is reduced by one half compared to undoped ZnO, which shows that the conductivity decreases due to Cl doping. However, one has to be very careful for avoiding an overinterpretation of impedance data, because the grain-boundary influence can significantly differ from sample to sample. This can also be seen in Figure S6b (Supporting Information File 1). For higher Cl content, when the tendency for the formation of separated particles is more pronounced (Figure 3), the resistivity raises due to the enhanced portion of grain boundaries.

**Figure 7:**
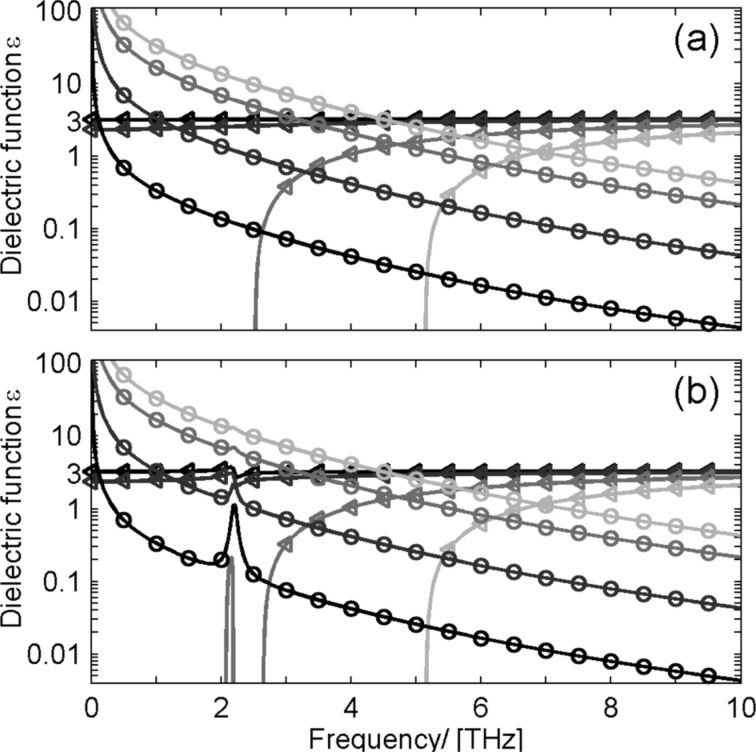
Theoretical model for dielectric function ε(w) for different carrier concentrations *N* = 1 × 10^14^ (black lines), *N* = 1 × 10^15^ (dark gray lines), *N* = 5 × 10^15^ (gray lines), and *N* = 1 × 10^16^ (light gray lines). The triangles mark the real parts ε_1_ and the circles the imaginary parts ε_2_; (a) Drude model; (b) Drude–Lorentz model with a resonance at 2 THz.

To get further information about the influence of doping on the conductivity, measurements of the complex dielectric function in the THz frequency range were performed. Time-domain THz spectroscopy [56–57] is a method to investigate the transmission and/or reflection of a sample in the THz frequency range. The transmitted electric field is directly sampled in the time domain, which provides amplitude and phase information of the transmission spectrum. A comparison with a reference measurement allows for the calculation of the complex dielectric function ε(w) of the sample material [82–83]. Free-carrier absorption at low carrier concentrations and IR-active lattice vibrations determine the complex response function in the THz frequency range [84]. A theoretical approach for modelling the complex dielectric response is the Drude model, see Figure 7a.

**Figure 8:**
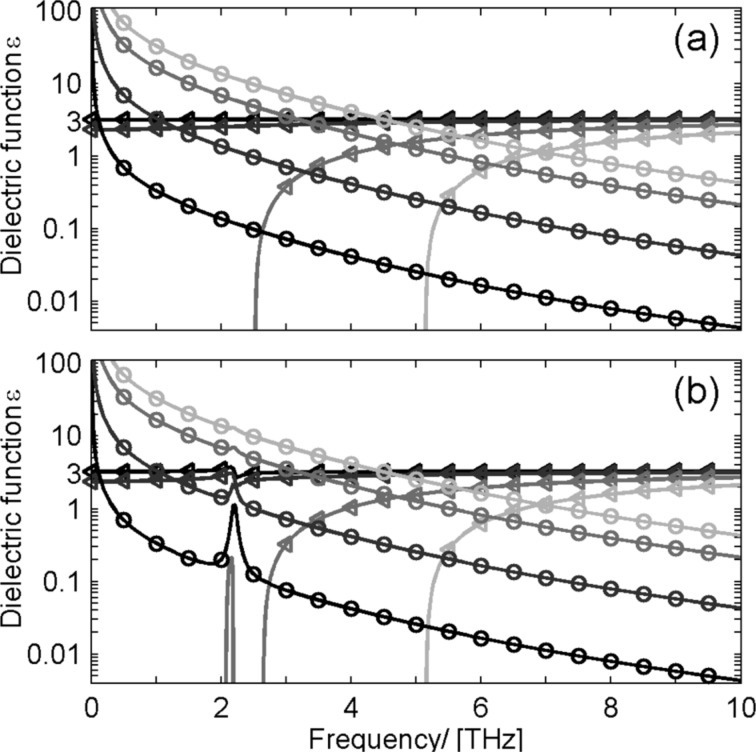
Measured dielectric function of ZnO_1−_*_x_*Cl*_x_* (a) real part; (b) imaginary part with *x* = 0.0% (black lines), 1.4% (dark gray lines), 1.8% (gray lines) and 2.5% (light gray lines); An infrared-active phonon at about about 2 THz is scaling in intensity with increasing Cl concentration.

The Drude model only takes into account free carriers and describes the meta-like background absorption of free carriers in the material. The actual shape of the Drude function strongly depends on the square of the plasma frequency, which is proportional to the number *N* of free carriers. Additional phonon oscillation are introduced as Lorentz oscillators (see Figure 7b). For nanostructured materials the Drude model has to be modified [84–85]. However, for a first step of interpretation of the experimental data the most salient changes with carrier densities are already visible in the Drude model. Figure 8 shows the real and imaginary part of ε for the measured ZnO_1−_*_x_*Cl*_x_* pellets obtained from time-domain THz spectroscopy. Two features are most prominent. First there is a phonon resonance at about 2 THz which is strongly depending in intensity on the Cl concentration. It is not yet clear which lattice mode gives rise to this IR-active mode. Low-frequency Raman measurements have shown that there is no Raman-active mode at a corresponding Raman shift of about 67 cm^−1^ observable. Second the value of ε_1_ is nearly the same for all ZnO_1−_*_x_*Cl*_x_*. Compared to Figure 7a we conclude that the detected change in the number of free carriers is smaller than in the theoretical model. However, it should be noted, that any change in the Drude response of the free carriers in this frequency range is strongly masked by the contribution of the Cl-induced phonon mode.

### Bromine- and iodine-containing ZnO materials

Using the precursors **2a,b** containing iodine and bromine analogous experiments were performed. Taking the example of [IMe_3_Zn_4_(O*t*-Bu)_4_] (**2a**), the influence of the decomposition temperature on the composition of ZnO_1−_*_x_*I*_x_* materials is demonstrated. The iodine content decreases strongly with increasing decomposition temperature (Figure S7, Supporting Information File 1). ZnO_1−_*_x_*I*_x_* prepared at 200 °C exhibits an iodine content of 8.8%, the sample prepared at 350 °C only of 0.5%. Probably the precursor reacts with volatile compounds such as iodine alkyls or elemental iodine. ZnO_1−_*_x_*Br*_x_* can be prepared analogously via thermal decomposition of [BrMe_3_Zn_4_(O*t*-Bu)_4_] (**2b**) (Figure S8, Supporting Information File 1).

## Conclusion

Starting from well-known ZnO precursors with 'Zn_4_O_4_' heterocubane framework, we showed that it is possible to introduce one single Hal–Zn entity (Hal = I, Br, Cl). These compounds have been proven to be novel single-source precursors for halogen-doped ZnO materials. The stability of the Hal@ZnO materials against thermal loss of Hal depends on the difference in ionic radius of O^2−^ compared to Hal^−^. Cl@ZnO materials are stable at temperatures below 500 °C, and nanocrystalline powders differing in Cl content could be prepared. It was seen that the presence of Cl leads to a widening of the ZnO lattice in *a*,*b*-direction, and as a consequence anisotropic nanoparticles with preferential growth in *c*-direction were obtained. The latter result represents an interesting perspective to understand and to control anisotropic particle growth.

However, the main focus of the current study was to prove the successful incorporation of Cl, and in particular its positioning on the lattice positions of oxygen (Cl_O_•), which leads to n-doping and opens up a perspective to use the materials as potential TCO materials in the future. Although it could be proven by several methods, that the desired materials were obtained, due to the current, powder-like nature, the electronic properties are yet dominated by grain boundary effects. Therefore, future research in this field will address the synthesis of thin films minimizing the amount of grain boundaries. Furthermore, we could not yet realize a F-containing precursor. F@ZnO materials are also expected to be interesting TCO candidates and potential ITO substitutes.

## Supporting Information

File 1 Additional experimental data.

## References

[1] Ginley D S, Bright C (2000). MRS Bull.

[2] Gust D, Moore T A, Moore A L (2009). Acc Chem Res.

[3] Habas S E, Platt H A S, van Hest M F A M, Ginley D S (2010). Chem Rev.

[4] Liu H Y, Avrutin V, Izyumskaya N, Özgür Ü, Morkoc H (2010). Superlattices Microstruct.

[5] Hecht D S, Hu L, Irvin G (2011). Adv Mater.

[6] Kim K S, Zhao Y, Jang H, Lee S Y, Kim J M, Kim K S, Ahn J-H, Kim P, Choi J-Y, Hong B H (2009). Nature.

[7] Bae S, Kim H, Lee Y, Xu X, Park J-S, Zheng Y, Balakrishnan J, Lei T, Kim H R, Song Y I (2010). Nat Nanotechnol.

[8] Wu Z, Chen Z, Du X, Logan J M, Sippel J, Nikolou M, Kamaras K, Reynolds J R, Tanner D B, Hebard A F (2004). Science.

[9] De S, Higgins T M, Lyons P E, Doherty E M, Nirmalraj P N, Blau W J, Boland J J, Coleman J N (2009). ACS Nano.

[10] Perelaer J, de Gans B-J, Schubert U S (2006). Adv Mater.

[11] Forrest S R (2004). Nature.

[12] Minami T (2005). Semicond Sci Technol.

[13] Fortunato E, Ginley D, Hosono H, Paine D C (2007). MRS Bull.

[14] Lewis B G, Paine D C (2000). MRS Bull.

[15] Minami T (2008). Thin Solid Films.

[16] Minami T (2008). Thin Solid Films.

[17] Klingshirn C (2007). Phys Status Solidi B.

[18] Klingshirn C (2007). ChemPhysChem.

[19] Maksimov O (2010). Rev Adv Mater Sci.

[20] Liu L, Xu J, Wang D, Jiang M, Wang S, Li B, Zhang Z, Zhao D, Shan C-X, Yao B (2012). Phys Rev Lett.

[21] Yoo J, Lee J, Kim S, Yoon K, Park I J, Dhungel S K, Karunagaran B, Mangalaraj D, Yi J (2005). Thin Solid Films.

[22] Agura H, Suzuki A, Matsushita T, Aoki T, Okuda M (2003). Thin Solid Films.

[23] Yakuphanoglu F, Caglar Y, Ilican S, Caglar M (2007). Physica B.

[24] Yoon H S, Lee K S, Lee T S, Cheong B, Choi D K, Kim D H, Kim W M (2008). Sol Energy Mater Sol Cells.

[25] Sanchez-Juarez A, Tiburcio-Silver A, Ortiz A (1998). Sol Energy Mater Sol Cells.

[26] Cui J B, Soo Y C, Chen T P, Gibson U J (2008). J Phys Chem C.

[27] Fan J, Shavel A, Zamani R, Fábrega C, Rousset J, Haller S, Güell F, Carrete A, Andreu T, Arbiol J (2011). Acta Mater.

[28] Lupan O, Pauporté T, Chow L, Viana B, Pellé F, Ono L K, Cuenya B R, Heinrich H (2010). Appl Surf Sci.

[29] Rousset J, Saucedo E, Herz K, Lincot D (2011). Prog Photovoltaics.

[30] Rousset J, Saucedo E, Lincot D (2009). Chem Mater.

[31] Lu J G, Fujita S, Kawaharamura T, Nishinaka H, Kamada Y, Ohshima T, Ye Z Z, Zeng Y J, Zhang Y Z, Zhu L P (2007). J Appl Phys.

[32] Lu J G, Ye Z Z, Zeng Y J, Zhu L P, Wang L, Yuan J, Zhao B H, Liang Q L (2006). J Appl Phys.

[33] Yousefi R, Jamah-Sheini F (2012). Ceram Int.

[34] Lee J-c, Subramaniam N G, Lee J-w, Lee J-c, Kang T-w (2013). Phys Status Solidi A.

[35] Burda C, Chen X, Narayanan R, El-Sayed M A (2005). Chem Rev.

[36] Yin Y, Alivisatos A P (2005). Nature.

[37] Mohanan J L, Arachchige I U, Brock S L (2005). Science.

[38] Livage J, Henry M, Sanchez C (1988). Prog Solid State Chem.

[39] Niederberger M (2007). Acc Chem Res.

[40] Norris D J, Efros A L, Erwin S C (2008). Science.

[41] Barrelet C J, Wu Y, Bell D C, Lieber C M (2003). J Am Chem Soc.

[42] Cumberland S L, Hanif K M, Javier A, Khitrov G A, Strouse G F, Woessner S M, Yun C S (2002). Chem Mater.

[43] Polarz S, Roy A, Merz M, Halm S, Schröder D, Schneider L, Bacher G, Kruis F E, Driess M (2005). Small.

[44] Veith M, Mathur S, Lecerf N, Huch V, Decker T, Beck H P, Eiser W, Haberkorn R (2000). J Sol-Gel Sci Technol.

[45] Singh S, Chaturvedi J, Bhattacharya S (2014). RSC Adv.

[46] Aksu Y, Frasca S, Wollenberger U, Driess M, Thomas A (2011). Chem Mater.

[47] Polarz S, Pueyo C L, Krumm M (2010). Inorg Chim Acta.

[48] Polarz S, Roy A, Lehmann M, Driess M, Kruis F E, Hoffmann A, Zimmer P (2007). Adv Funct Mater.

[49] Polarz S, Strunk J, Ischenko V, van den Berg M W E, Hinrichsen O, Muhler M, Driess M (2006). Angew Chem, Int Ed.

[50] Polarz S, Orlov A V, van den Berg M W E, Driess M (2005). Angew Chem, Int Ed.

[51] Orlov A, Roy A, Lehmann M, Driess M, Polarz S (2007). J Am Chem Soc.

[52] Dreher M A, Krumm M, Lizandara-Pueyo C, Polarz S (2010). Dalton Trans.

[53] Polarz S, Orlov A, Hoffmann A, Wagner M R, Rauch C, Kirste R, Gehlhoff W, Aksu Y, Driess M, van den Berg M W E (2009). Chem Mater.

[54] Lehr D, Luka M, Wagner M R, Bügler M, Hoffmann A, Polarz S (2012). Chem Mater.

[55] Lizandara-Pueyo C, van den Berg M W E, De Toni A, Goes T, Polarz S (2008). J Am Chem Soc.

[56] Gebs R, Klatt G, Janke C, Dekorsy T, Bartels A (2010). Opt Express.

[57] Klatt G, Gebs R, Schäfer H, Nagel M, Janke C, Bartels A, Dekorsy T (2011). IEEE J Sel Top Quantum Electron.

[58] Pueyo C L, Siroky S, Landsmann S, van den Berg M W E, Wagner M R, Reparaz J S, Hoffmann A, Polarz S (2010). Chem Mater.

[59] Dilger S, Lizandara-Pueyo C, Krumm M, Polarz S (2012). Adv Mater.

[60] Lizandara-Pueyo C, Siroky S, Wagner M R, Hoffmann A, Reparaz J S, Lehmann M, Polarz S (2011). Adv Funct Mater.

[61] Polarz S, Orlov A V, Schüth F, Lu A H (2007). Chem – Eur J.

[62] Polarz S, Neues F, van den Berg M W E, Grünert W, Khodeir L (2005). J Am Chem Soc.

[63] Ischenko V, Polarz S, Grote D, Stavarache V, Fink K, Driess M (2005). Adv Funct Mater.

[64] Driess M, Merz K, Rell S (2000). Eur J Inorg Chem.

[65] Bratsch S G (1985). J Chem Educ.

[66] Krumm M, Pueyo C L, Polarz S (2010). Chem Mater.

[67] Lizandara-Pueyo C, Morant-Miñana M C, Wessig M, Krumm M, Mecking S, Polarz S (2012). RSC Adv.

[68] Polarz S (2011). Adv Funct Mater.

[69] Burstein E (1954). Phys Rev.

[70] Moss T S (1954). Proc Phys Soc, London, Sect B.

[71] Lautenschlaeger S, Eisermann S, Haas G, Zolnowski E A, Hofmann M N, Laufer A, Pinnisch M, Meyer B K, Wagner M R, Reparaz J S (2012). Phys Rev B.

[72] Schirra M, Schneider R, Reiser A, Prinz G M, Feneberg M, Biskupek J, Kaiser U, Krill C E, Thonke K, Sauer R (2008). Phys Rev B.

[73] Meyer B K, Sann J, Lautenschläger S, Wagner M R, Hoffmann A (2007). Phys Rev B.

[74] Wagner M R, Callsen G, Reparaz J S, Schulze J-H, Kirste R, Cobet M, Ostapenko I A, Rodt S, Nenstiel C, Kaiser M (2011). Phys Rev B.

[75] Kirste R, Mohn S, Wagner M R, Reparaz J S, Hoffmann A (2012). Appl Phys Lett.

[76] Sahoo S, Sharma G L, Katiyar R S (2012). J Raman Spectrosc.

[77] Thakur J S, Haddad D, Naik V M, Naik R, Auner G W, Lu H, Schaff W J (2005). Phys Rev B.

[78] Demangeot F, Pinquier C, Frandon J, Gaio M, Briot O, Maleyre B, Ruffenach S, Gil B (2005). Phys Rev B.

[79] Reparaz J S, Muniz L R, Wagner M R, Goñi A R, Alonso M I, Hoffmann A, Meyer B K (2010). Appl Phys Lett.

[80] Venkatesh P S, Ramakrishnan V, Jeganathan K (2012). CrystEngComm.

[81] Díaz-Flores L L, Ramírez-Bon R, Mendoza-Galván A, Prokhorov E, González-Hernández J (2003). J Phys Chem Solids.

[82] Duvillaret L, Garet F, Coutaz J-L (1999). Appl Opt.

[83] Dorney T D, Baraniuk R G, Mittleman D M (2001). J Opt Soc Am A.

[84] Lloyd-Hughes J, Jeon T-I (2012). J Infrared, Millimeter, Terahertz Waves.

[85] Kužel P, Němec H (2014). J Phys D: Appl Phys.

