# Correction: A single-source precursor route to anisotropic halogen-doped zinc oxide particles as a promising candidate for new transparent conducting oxide materials

**DOI:** 10.3762/bjnano.6.239

**Published:** 2015-12-08

**Authors:** Daniela Lehr, Markus R Wagner, Johanna Flock, Julian S Reparaz, Clivia M Sotomayor Torres, Alexander Klaiber, Thomas Dekorsy, Sebastian Polarz

**Affiliations:** 1 Department of Chemistry, University of Konstanz, 78457 Konstanz, Germany; 2 ICN2 Catalan Institute of Nanoscience and Nanotechnology, Campus UAB, 08193 Bellaterra (Barcelona), Spain; 3 Department of Physics, University of Konstanz, 78457 Konstanz, Germany; 4 Catalan Institute of Research and Advanced Studies (ICREA), Barcelona 08010, Spain

**Keywords:** chemical doping, metal oxides, semiconductor nanoparticles, single-source precursors

In the original article an incorrect graphic was displayed for Figure 8. The correct form of Figure 8 is as follows:

**Figure 1:**
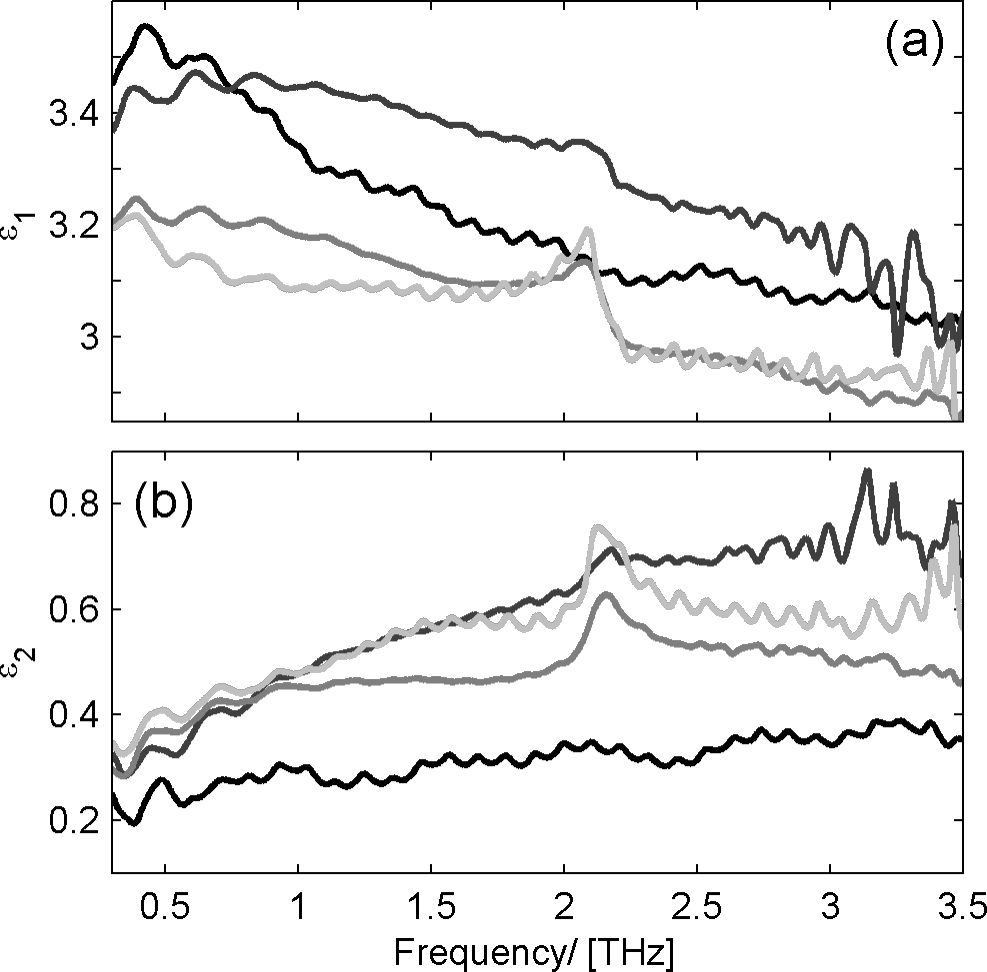
**Figure 8 in the original article:** Measured dielectric function of ZnO_1−_*_x_*Cl*_x_* (a) real part; (b) imaginary part with *x* = 0.0% (black lines), 1.4% (dark gray lines), 1.8% (gray lines) and 2.5% (light gray lines); An infrared-active phonon at about 2 THz is scaling in intensity with increasing Cl concentration.

