# Supplementary material for: A single-source precursor route to anisotropic halogen-doped zinc oxide particles as a promising candidate for new transparent conducting oxide materials
Source: Beilstein J Nanotechnol. 2015 Nov 18;6:2161–72. doi: 10.3762/bjnano.6.222 (PMC4660940; doi:10.3762/bjnano.6.222)
Supplement: File 1 — Additional experimental data. [file Beilstein_J_Nanotechnol-06-2161-s001.pdf]

Supporting Information

for

A single-source precursor route to anisotropic halogen-doped zinc oxide particles as a promising candidate for new transparent conducting oxide materials

Daniela Lehr[1], Markus R. Wagner[2], Johanna Flock[3], Julian S. Reparaz[2],

Clivia M. Sotomayor Torres[2,4], Alexander Klaiber[1], Thomas Dekorsy[3] and Sebastian Polarz*[1]

Address: [1]Department of Chemistry, University of Konstanz, 78457 Konstanz, Germany; [2]ICN2 Catalan Institute of Nanoscience and Nanotechnology, Campus UAB, 08193 Bellaterra (Barcelona), Spain; [3]Department of Physics, University of Konstanz, 78457 Konstanz, Germany and [4]Catalan Institute of Research and Advanced Studies (ICREA), Barcelona 08010, Spain

Email: Sebastian Polarz* - sebastian.polarz@uni-konstanz.de

*Corresponding author

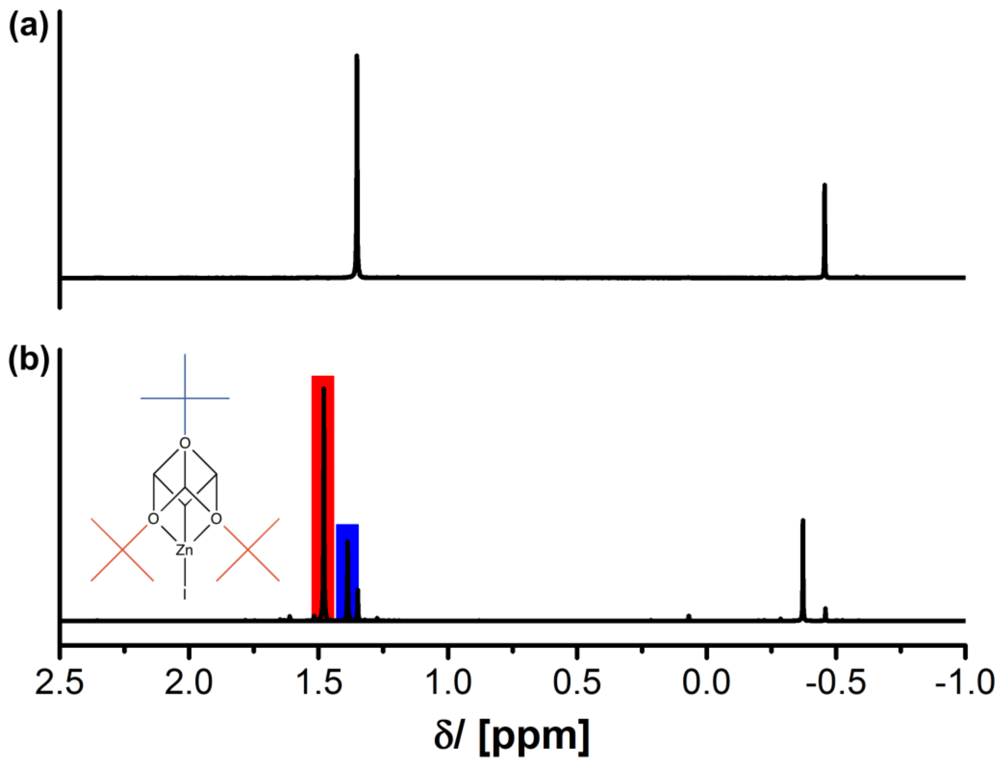
(a)
(b)
O
O
O
Zn
2.5
2.0
1.5
1.0
0.5
0.0
-0.5
-1.0
δ/ [ppm]

(c)

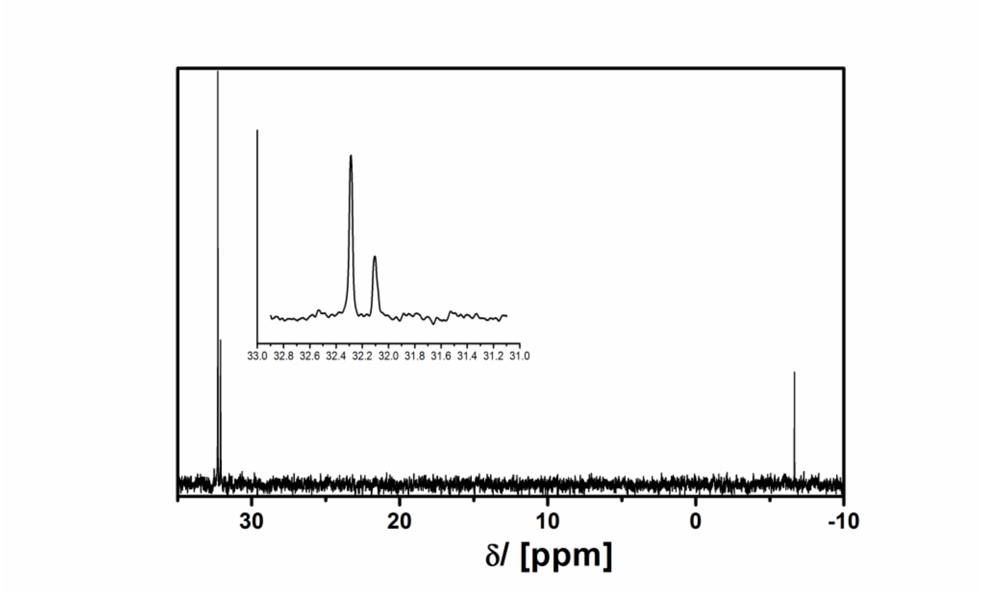
33.0 32.8 32.6 32.4 32.2 32.0 31.8 31.6 31.4 31.2 31.0
30
20
10
0
-10
δ/ [ppm]

(d)

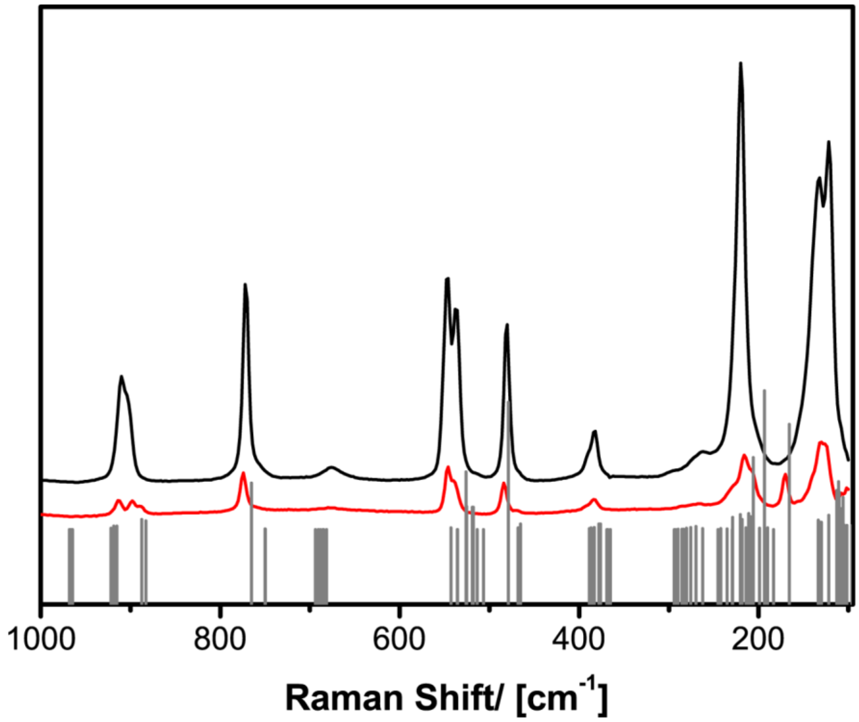

Figure S1: Additional analytical data for the molecular precursor compound **2a**. ^{1}H NMR (400 MHz, $CDCl_3$) spectrum of the starting compound **1** [MeZnO*t*-Bu]$_4$ (a) and the monosubstituted iodo-compound **2a** [I(CH_3)$_3$Zn(O*t*-Bu)$_4$]. (c) ^{13}C NMR (400 MHz, $CDCl_3$) spectrum of the monosubstituted iodo-compound **2a** [I(CH_3)$_3$Zn$_4$(O*t*-Bu)$_4$]. (d) FT-Raman spectra of [MeZnO*t*-Bu]$_4$ (black) and [I(CH_3)$_3$Zn$_4$(O*t*-Bu)$_4$] (**2a**) (red). Grey bars: DFT simulated vibration spectra of **2a**.

(a)

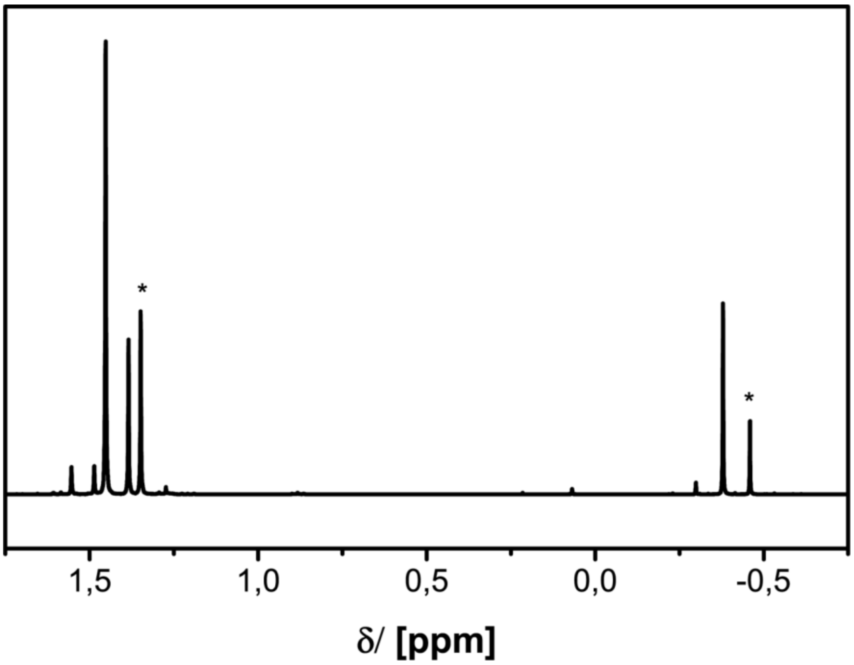

(b)

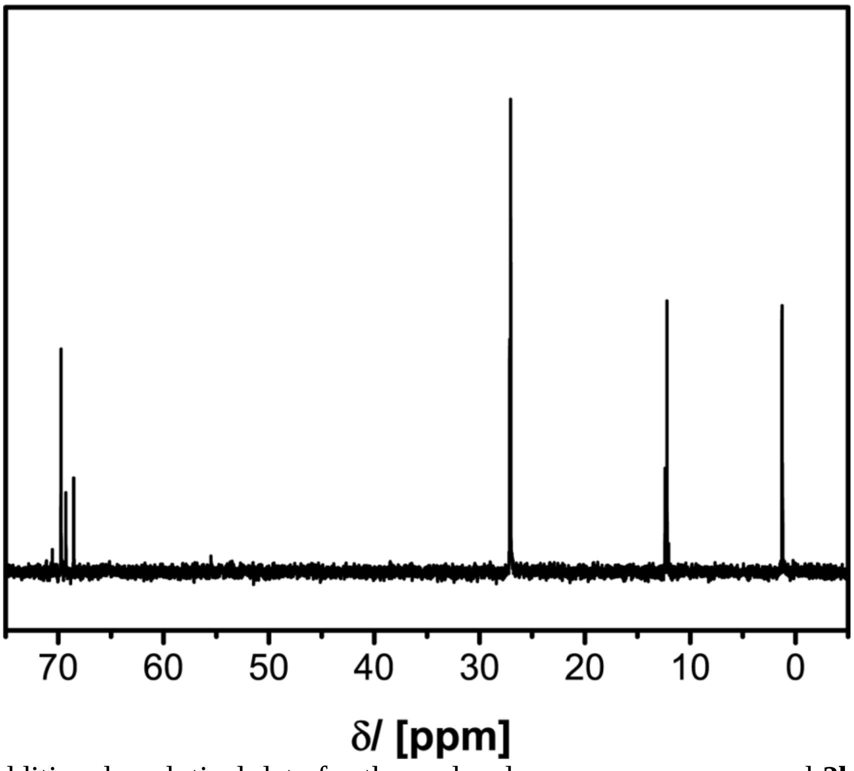

Figure S2: Additional analytical data for the molecular precursor compound **2b**. (a) ^{1}H NMR (400 MHz, $CDCl_3$) spectrum of the mono-substituted bromo-compound **2b** $[Br(CH_3)_3Zn_4(O\textit{t}\text{-Bu})_4]$ (*residual starting compound $[MeZnO\textit{t}\text{-Bu}]_4$). (b) ^{13}C NMR (400 MHz, $CDCl_3$) spectrum of the mono-substituted bromo-compound **2b** $[Br(CH_3)_3Zn_4(O\textit{t}\text{-Bu})_4]$.

(a)

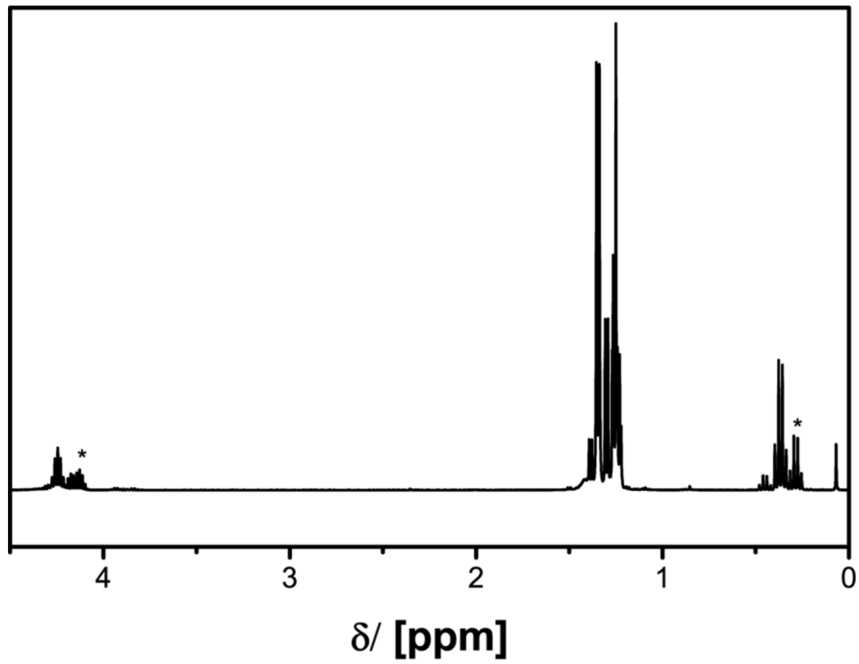

(b)

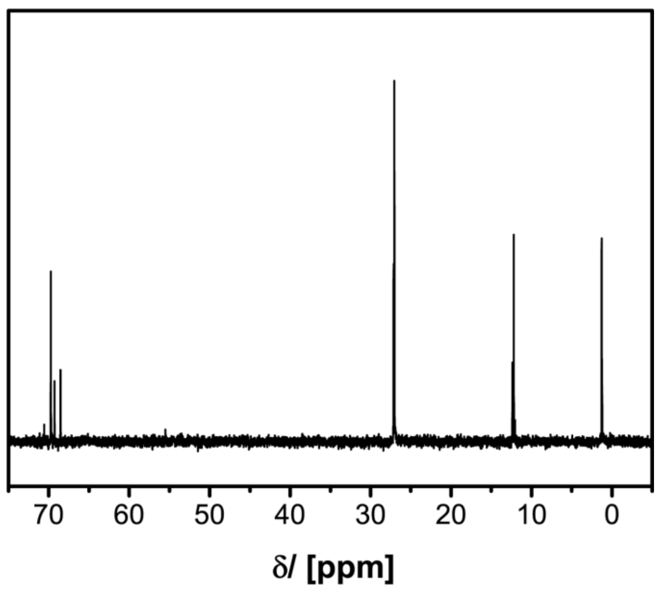

(c)

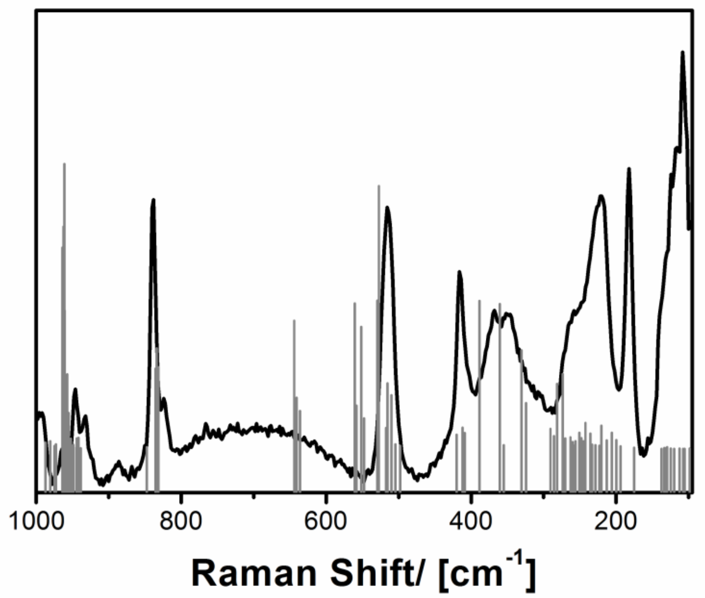

(d)

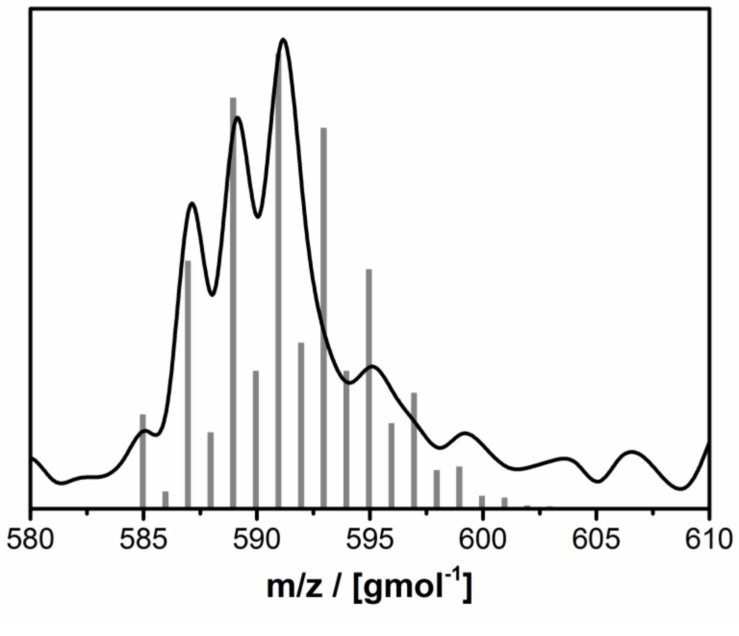

Figure S3: Additional analytical data for the molecular precursor compound **2c**. (a) ^{1}H NMR (400 MHz, $CDCl_3$) spectrum of the monosubstituted chloro-compound $[Cl(Et)_3Zn_4(OiPr)_4]$ (**2c**) (*residual starting compound $[EtZnOiPr]_4$). (b) ^{13}C NMR (400 MHz, $CDCl_3$) spectrum of the monosubstituted chloro-compound $[Cl(Et)_3Zn_4(OiPr)_4]$ (**2c**). (c) FT-Raman of $[Cl(Et)_3Zn_4(OiPr)_4]$; experimental spectrum: black graph; spectrum simulated using DFT calculations: grey bars. EI-MS data (black graph: experimental signal) and calculated signal (grey bars) for the fragment $[Cl(Et)_2Zn(OiPr)_4]^+$ (m/z = 591.5).

(a)

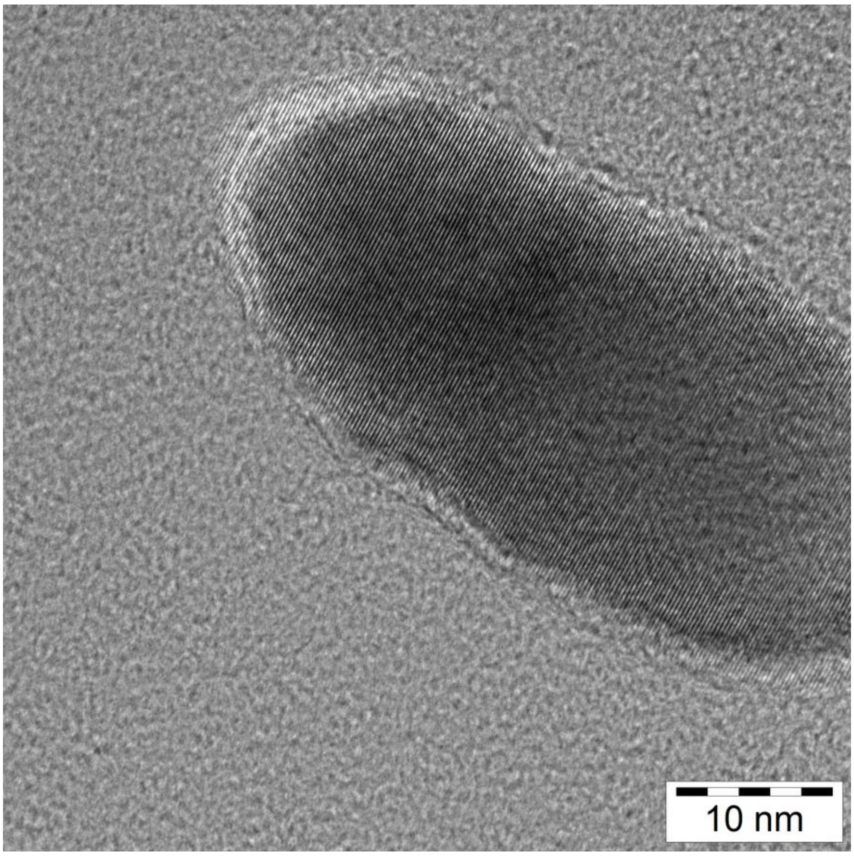

(b)

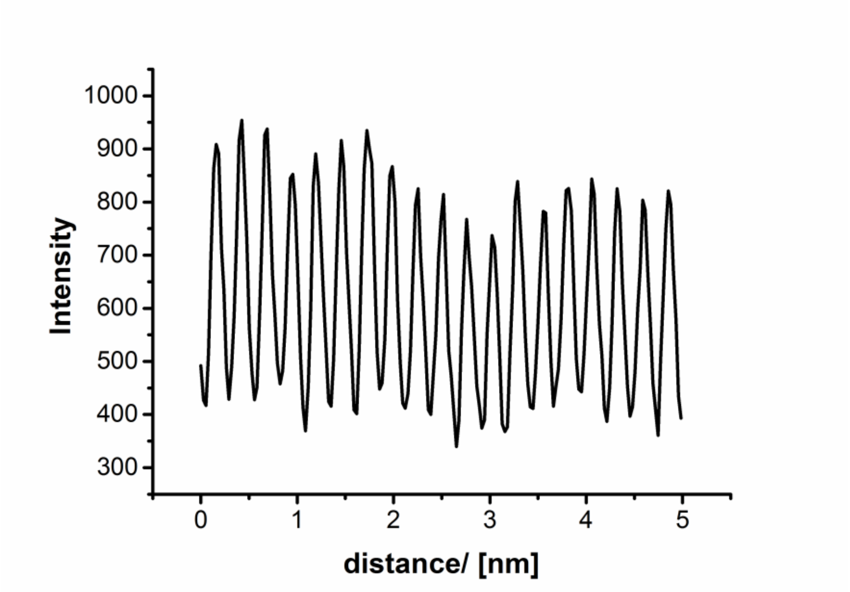

Figure S4: (a) HRTEM evaluation of $ZnO_{1-x}Cl_x$. (b) Evaluation of the lattice plane distances along *c*-direction from HR-TEM micrograph.

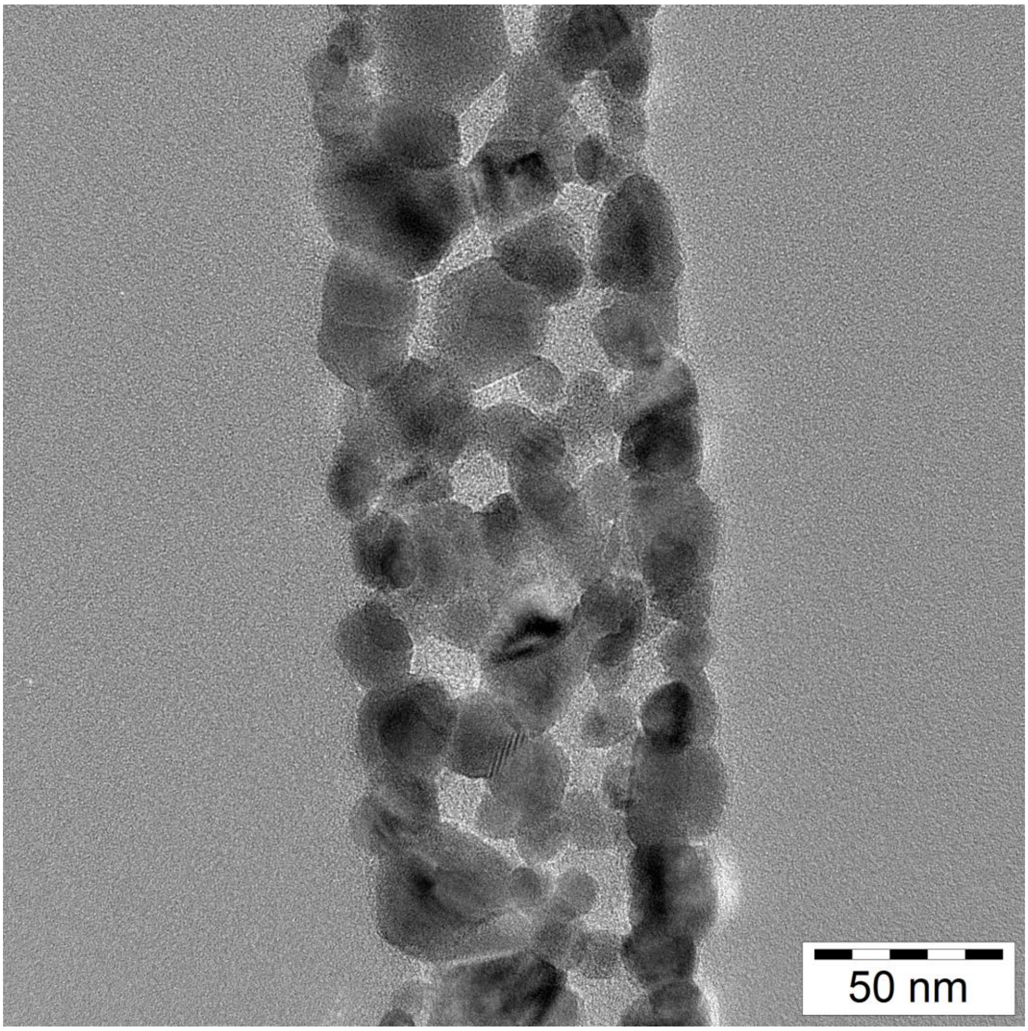

Figure S5: TEM micrograph of ZnO particles prepared from $[EtZnOiPr]_4$ as a precursor.

(a)

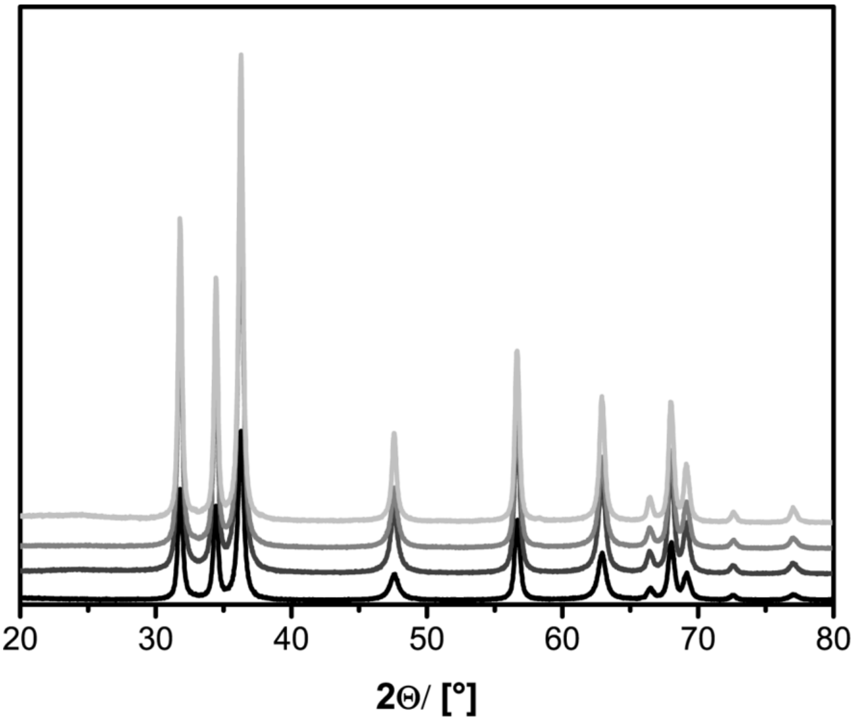

Composition and crystallite size of $ZnO_{1-x}Cl_x$

x in $ZnO_{1-x}Cl_x$	$D_{cryst[110]}$ [nm]
0.0	21.6
1.4	25.1
1.8	27.2
2.5	37.1

The crystallite size was calculated from the (110) diffraction signal using Scherrer equation.

(b)

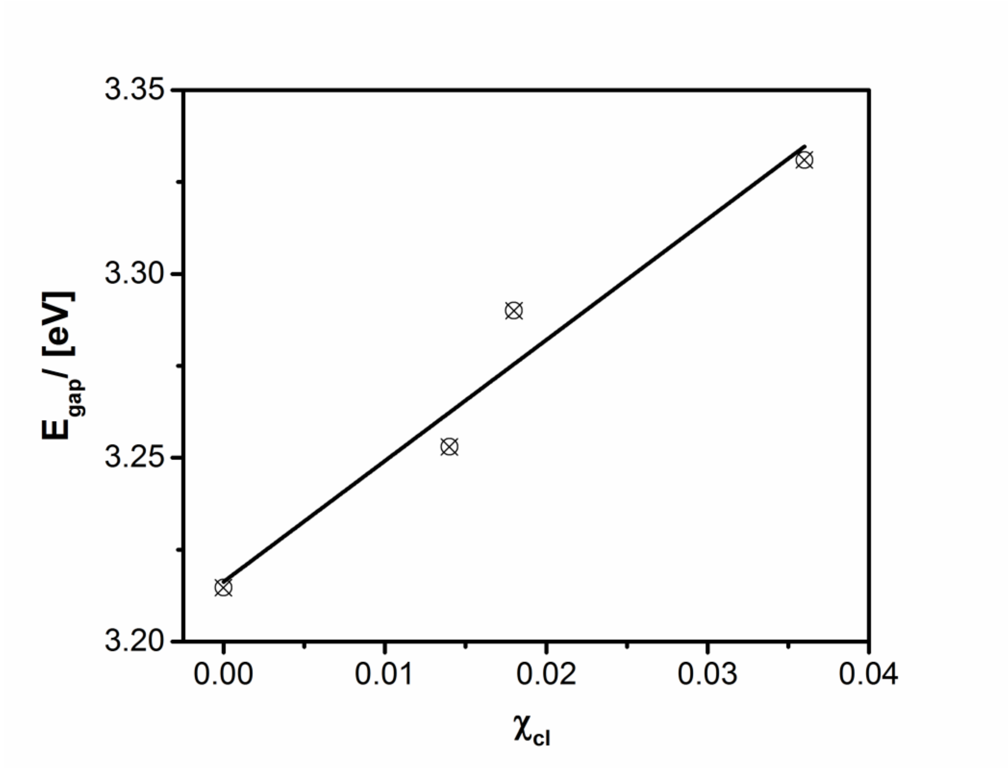

(c)

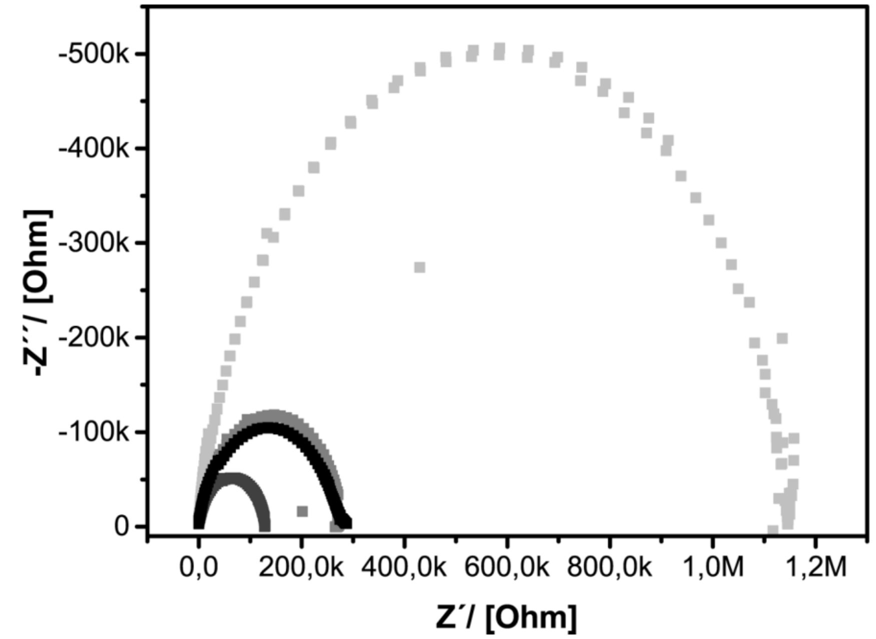

Figure S6: $ZnO_{1-x}Cl_x$ materials with different doping concentrations. (a) PXRD of $ZnO_{1-x}Cl_x$ materials synthesized from precursor mixtures of $[Et_3Cl_1Zn_4(OiPr)_4]$ (**2c**) and $[EtZnOiPr]_4$: $x = 0.0\%$ (black), 1.4% (dark grey), 1.8% (grey) and 2.5% (light grey). (b) Correlation of E_{gap} determined from optical diffuse reflectance spectra and the mole fraction of chlorine. (c) Impedance spectra of $ZnO_{1-x}Cl_x$ (Nyquist Plot): $x = 0.0\%$ (black), 1.4% (dark grey), 1.8% (grey) and 2.5% (light grey).

(a)

T [°C]	x in $ZnO_{1-x}I_x$	$D_{cryst\ [110]}$ [nm]
200	0.088	7.7
250	0.024	15.1
300	0.007	15.7
350	0.005	19.9
350 (ZnO reference)	0.000	18.4

(b)

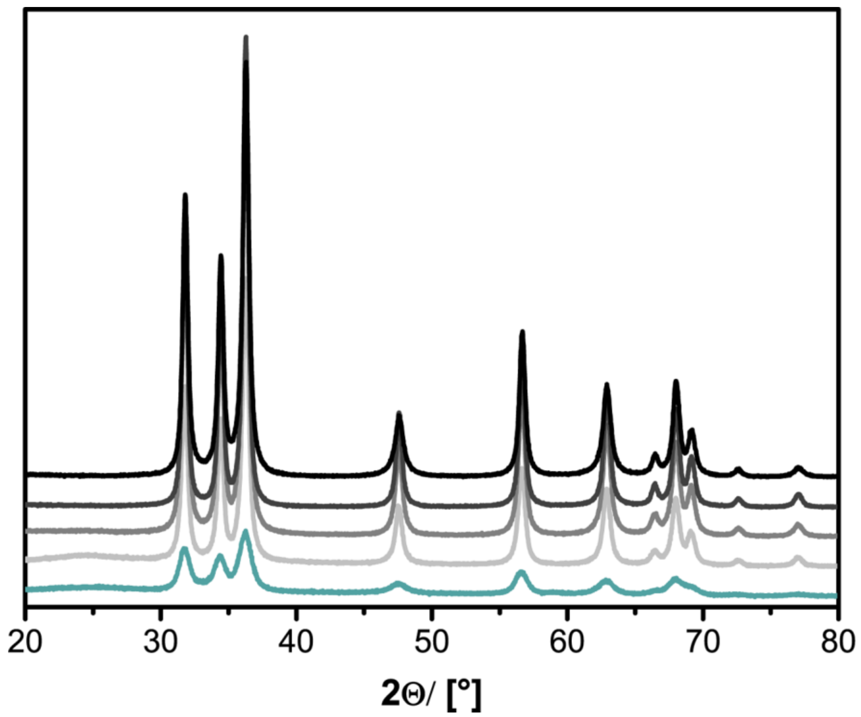

(c)

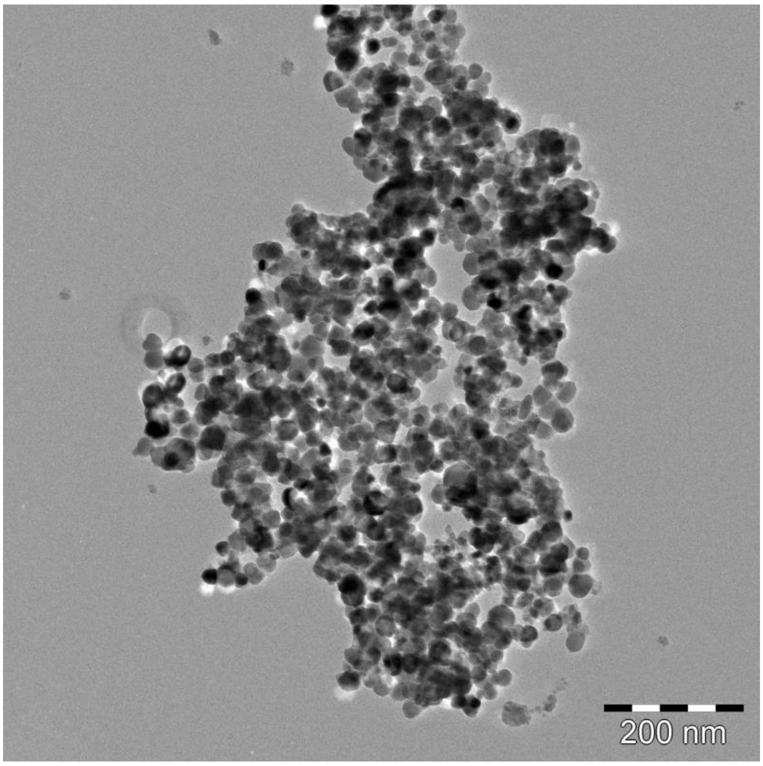

Figure S7: $ZnO_{1-x}I_x$ materials prepared from precursor **2a**. (a) $ZnO_{1-x}I_x$ synthesized from **2a** at different decomposition temperatures. (b) PXRD of $ZnO_{1-x}I_x$ materials synthesized at different temperatures from $[IMe_3Zn(Ot\text{-}Bu)_4]$: 200 °C (blue), 250 °C (light grey), 300 °C (grey) and 350 °C (darkgrey). Black: ZnO reference (prepared from $[MeZnOt\text{-}Bu]_4$ at 350 °C). (c) TEM micrograph of $ZnO_{1-x}I_x$ particles ($x = 0.005$).

(a)

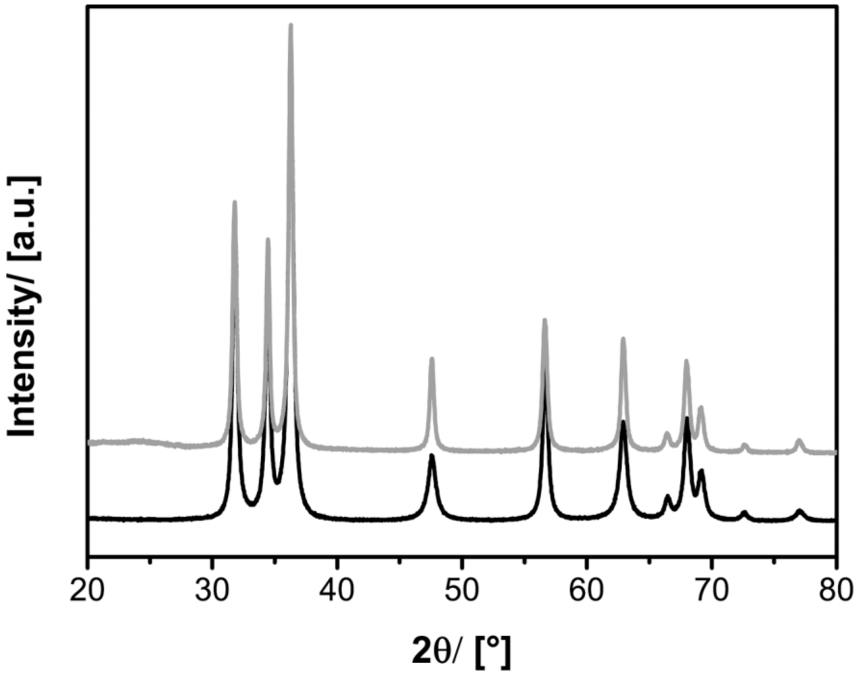

(b)

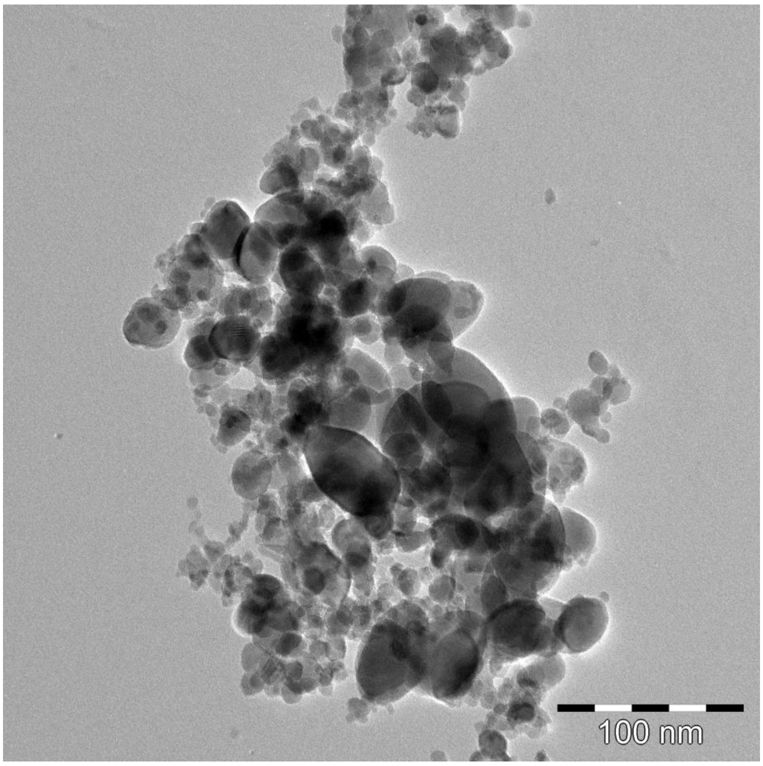

Figure S8: $ZnO_{1-x}Br_x$ material prepared from precursor **2b**. (a) PXRD of $ZnO_{1-x}Br_x$ (x = 0.022) and ZnO reference (black) prepared at 350 °C. (b) TEM micrograph of $ZnO_{1-x}Br_x$ particles.
